# A Dual-Adaptive Sage–Husa Kalman Filter with Model Matching Check for GNSS/INS-Integrated Navigation

**DOI:** 10.3390/s26154925

**Published:** 2026-08-04

**Authors:** Mingduan Zhou, Shiqi Lin, Peng Yan, Qiao Song, Lu Qin, Shufa Li, Lingyan Kong, Guanxiu Wu, Zihan Zhou, Qianlong Xie, Qingfeng Zeng, Yuhan Qin

**Affiliations:** 1 School of Geomatics and Urban Spatial Informatics, Beijing University of Civil Engineering and Architecture, Beijing 102616, China; 2108160125006@stu.bucea.edu.cn (P.Y.); 2108160124002@stu.bucea.edu.cn (Q.S.); 2108570424058@stu.bucea.edu.cn (L.Q.); 2108570425026@stu.bucea.edu.cn (S.L.); 2108160225010@stu.bucea.edu.cn (G.W.); 2108160225013@stu.bucea.edu.cn (Z.Z.); 2108160123008@stu.bucea.edu.cn (Q.X.); 2108570023128@stu.bucea.edu.cn (Y.Q.); 2 Beijing Institute of Surveying and Mapping, Beijing 100038, China; konglingyan@bism.cn (L.K.); zengqingfeng@bism.cn (Q.Z.)

**Keywords:** GNSS/INS integrated navigation, Sage–Husa filter, extended Kalman filter, adaptive filter, model mismatch detection, measurement noise estimation

## Abstract

**Highlights:**

**What are the main findings?**

- A model-adaptation-aware dual-adaptive Sage–Husa filtering algorithm is proposed. Through a mechanism combining global recursive updating based on the forgetting factor and local response based on an adaptive sliding window, rapid tracking and stable estimation of time-varying measurement noise are achieved.
- A model matching test and an upper-bound detection module for the measurement noise covariance are introduced to effectively distinguish measurement noise disturbances from model mismatch errors. Under high-dynamic conditions, instantaneous GNSS interference, and continuous weak-observation scenarios, positioning accuracy, attitude stability, and filtering convergence robustness are significantly improved.

**What are the implications of the main findings?**

- The proposed dual-adaptive filtering algorithm can stably and accurately track time-varying measurement noise under high-dynamic conditions and complex observation environments, providing a reliable real-time noise adaptation solution for vehicle-mounted GNSS/INS integrated navigation systems.
- Through the model matching test and the covariance upper-bound detection module, noise disturbances and model mismatch errors can be effectively distinguished. This provides theoretical and engineering support for improving filter stability and robust-ness in complex environments, and can be extended to unmanned systems, intelligent transportation, and other high-dynamic navigation scenarios.

**Abstract:**

GNSS/INS integrated navigation combines the long-term stability of satellite positioning with the high-frequency continuity of inertial navigation, and has been widely adopted in vehicle navigation, unmanned systems, and intelligent transportation systems. However, in complex urban environments, GNSS measurement noise exhibits significant time-varying characteristics, while high-dynamic carrier motions may cause mismatches between the dynamic model and the actual motion state. These two factors jointly degrade the performance of conventional Sage–Husa (cSage–Husa) filters, resulting in distorted measurement noise estimation and over-adaptation. To address this issue, this paper proposes a dual-adaptive Sage–Husa filtering algorithm considering model matching. The proposed algorithm integrates a forgetting-factor-based global recursive estimation mechanism with an adaptive sliding window strategy. Furthermore, a model matching test and a measurement noise covariance upper-bound constraint are introduced to distinguish model mismatch errors from normal measurement noise variations, thereby improving filtering stability and noise estimation reliability. Experimental results demonstrate that, under initial high-dynamic motions and instantaneous GNSS interference conditions, the proposed algorithm reduces the up-channel RMS error by 8.5% compared with cSage–Husa. Under sustained GNSS degradation conditions, the north RMS error is reduced by 8.7% and 2.5% compared with EKF and cSage–Husa, respectively, while the up-channel RMS error is reduced by 15% compared with cSage–Husa and maintains comparable accuracy to EKF. The proposed algorithm effectively mitigates over-adaptation and convergence degradation caused by model mismatch, achieving a favorable balance between time-varying noise tracking capability and filtering stability for urban canyon and high-dynamic vehicle positioning.

## 1. Introduction

GNSS/INS integrated navigation has become a mainstream positioning solution for vehicle navigation, unmanned systems, and intelligent transportation applications by combining the long-term stability of GNSS with the high-frequency short-term continuity of INS [1,2]. However, in complex environments such as urban canyons, tree-covered roads, tunnels, and dense elevated-road networks, GNSS signals are prone to outages, attenuation, and multipath interference, resulting in measurement noise with pronounced non-Gaussian and time-varying characteristics [3]. Meanwhile, high-dynamic carrier motions, including rapid acceleration, deceleration, and steering, may violate the underlying motion-model assumptions and introduce significant model mismatch, leading to delayed filter convergence and increased attitude and velocity estimation errors. Consequently, GNSS/INS integrated navigation systems are simultaneously challenged by observation degradation and maneuver-induced model mismatch. Although existing studies have improved positioning continuity and observation utilization in obstructed environments through tightly coupled integration strategies and post-processing smoothing techniques [4,5], achieving an effective balance between adaptive tracking of time-varying noise and suppression of model-induced errors remains a challenging issue.

Regarding EKF-based integrated navigation algorithms, conventional methods rely on pre-specified fixed noise statistics, making them difficult to adapt to dynamically varying observation environments. As a result, problems such as degraded filtering accuracy, slow convergence, error accumulation, and even filter divergence may occur, which makes it difficult to satisfy the requirements for highly reliable navigation in complex environments [6]. To improve the performance of adaptive filtering, optimal adaptive factors have been constructed to balance the contributions of model information and observations, thereby providing a theoretical foundation for subsequent noise-adaptive methods [7]. In addition, one type of improved scheme directly uses the GNSS posterior covariance to drive the estimation of the measurement noise matrix. Specifically, the posterior covariance matrix obtained from GNSS single-point positioning or RTK solutions is directly used as the measurement noise matrix (R) of the integrated filter, so that the observation quality can be directly reflected. This scheme does not require an additional adaptive estimation module and is simple to implement with low computational cost. However, it still has obvious limitations. The GNSS posterior covariance mainly reflects the theoretical precision of the solution process and does not include the actual noise caused by external disturbances such as multipath effects and non-line-of-sight propagation, resulting in biased estimation of the true observation errors. Meanwhile, the GNSS solution update interval is usually much longer than the INS recursive update interval, which leads to a delayed response to time-varying measurement noise. In scenarios involving observation outage recovery or strong interference, an underestimated R may cause the filter to over-trust abnormal observations, thereby inducing filtering oscillation or even divergence [8,9]. Moreover, after observation outage recovery, the GNSS solution module generally requires a relatively long period to reconverge. This indicates that the measurement noise estimation of the integrated filter may be severely distorted during this stage, further increasing the risk of filtering oscillation or divergence. To address these problems, interference suppression methods under tightly coupled architectures have been proposed successively; however, the contradiction between time-varying noise and model mismatch has not yet been fully resolved [10,11].

In the context of robust filtering based on M-estimation, such methods have been widely applied in integrated navigation systems to suppress outliers and non-Gaussian noise. The fundamental idea is to perform adaptive weighting of observation residuals using robust kernel functions such as Huber, Cauchy, and Geman–McClure, or to further construct an equivalent measurement noise covariance matrix to reduce the weight of abnormal observations during filter updates, thereby mitigating their negative impact on state estimation results [12,13]. On this basis, the combination of recursive strategies and generalized M-estimation can further enhance the consistency of filtering estimation in nonlinear systems [14]. However, traditional M-estimation methods still exhibit certain limitations. Firstly, the key thresholds in robust kernel functions are typically set based on empirical experience, making it difficult to adaptively match the variations of time-varying noise and dynamic outliers. Secondly, such methods generally construct robust weighting functions based on observation residuals and adjust the contributions of different observations in filter updates through equivalent weight matrices or equivalent measurement noise covariance matrices. Essentially, these methods still focus on residual-level weighting of observations, and their capability for modeling the time-varying characteristics of measurement noise itself is relatively limited. Therefore, in complex scenarios where the noise statistics are unknown and rapidly changing with the environment, conventional M-estimation methods struggle to simultaneously achieve both outlier suppression and adaptive estimation of time-varying noise. In addition, M-estimation methods based on iterative optimization are susceptible to the non-convexity of the objective function, which can result in convergence to local optima, reduced filtering convergence speed, and increased state estimation bias. Under high-dynamic motion conditions or frequent observation interruptions, these methods may also excessively suppress valid observation information, leading to delayed state tracking and degraded navigation accuracy. Consequently, existing M-estimation-based robust filtering approaches remain limited in their ability to simultaneously realize adaptive noise estimation and outlier suppression in complex urban environments.

In the context of noise-adaptive estimation based on the Sage–Husa algorithm, the method achieves online recursive estimation of the noise covariance by introducing a forgetting factor, enabling the dynamic update of noise statistical parameters without relying on precise a priori information. This enhances the filter’s adaptability to time-varying noise statistics and has become an important approach for noise suppression in integrated navigation [15]. The method was originally proposed by Sage and Husa for adaptive filtering under unknown a priori statistical conditions [16] and has since been widely adopted in integrated navigation applications. The conventional Sage–Husa algorithm (hereafter referred to as cSage–Husa) assumes that the innovation sequence follows zero-mean Gaussian white noise driven solely by measurement noise, allowing real-time estimation of the measurement noise covariance from innovation statistics. In practical GNSS/INS integrated navigation systems. However, in practical GNSS/INS-integrated navigation systems, innovations are often affected by multiple factors, including measurement outliers, accumulated inertial errors, and model mismatches. In particular, during high-dynamic carrier motions, GNSS signal outages, or non-converged attitude phases, model errors can severely contaminate the innovations, causing deviations from ideal statistical assumptions and resulting in distorted noise estimation. Currently, multiple technical routes for optimizing the Sage–Husa filtering algorithm have been developed, focusing primarily on forgetting factor optimization, sliding window parameter tuning, robust weighting modifications, and enhancement of fault detection mechanisms. Previous studies have applied the Sage–Husa algorithm to SINS initial alignment, demonstrating that the forgetting factor effectively enhances innovation tracking, but the issue of model mismatch contamination remains insufficiently addressed [17]. Further research has been conducted to improve the conventional Sage–Husa algorithm, with applications including the suppression of random errors in fiber optic gyroscopes and other inertial sensors [18,19,20]. Narasimhappa et al. proposed an adaptive robust Sage–Husa filtering scheme targeting drift suppression in low-cost MEMS inertial sensors, effectively reducing navigation errors induced by sensor drift but not addressing model mismatch caused by high-dynamic carrier motions [21]. Xu et al. improved the Sage–Husa algorithm within a federated filter architecture, leveraging complementary multi-source navigation information to optimize the noise update logic, enhancing resilience to time-varying noise in multi-sensor fusion and improving navigation accuracy under complex conditions [22]. Wang et al. reconstructed the Sage–Husa measurement update using Huber robust estimation theory, employing robust equivalent weights to adaptively down-weight abnormal measurements, further integrating variable-coefficient sliding windows and fading filtering mechanisms to improve response to sudden noise changes and reduce convergence time after abrupt noise variations [23,24]. Liu et al. optimized the iterative formula from a numerical stability perspective by removing redundant terms that could cause negative definite covariance, ensuring positive definiteness of measurement and system noise matrices at minimal cost to positioning accuracy and preventing numerical divergence [25]. Song et al. integrated a multiple-model adaptive estimation framework with an improved Sage–Husa algorithm, using parallel multi-model selection to adapt to variable noise environments and enhance robustness under non-stationary noise conditions [26]. Lyu et al. developed a master–slave-nested dual-adaptive unscented Kalman filter architecture, with the master filter handling navigation state estimation and the slave filter using the improved Sage–Husa algorithm to iteratively update measurement noise covariance in real time [27]. Juston et al. optimized the Sage–Husa iteration logic in combination with error-state Kalman filtering and applied the improved algorithm to UWB indoor positioning scenarios to enhance stability in complex environments [28]. Jing et al. focused on covariance positive-definiteness constraints, adapting the improved Sage–Husa algorithm to the unscented filter framework and effectively enhancing overall GNSS/INS navigation stability [29]. Li proposed a dual forgetting-factor control strategy, dynamically adjusting the forgetting coefficients via a covariance matching criterion to mitigate filter divergence induced by a single forgetting factor [30]. Wang integrated dual-window fading weighting with fault detection modules, using innovation statistics to identify measurement faults and optimize navigation performance in challenging GNSS environments such as urban canyons [31]. Lin introduced dual regulation factors to separately control the noise update rate and fault detection thresholds, reconciling the conflicting requirements between online noise estimation and anomaly detection [32]. Lu abandoned the fixed window-length approach, dynamically adjusting sliding window sizes based on innovation fluctuations to achieve adaptive window parameter variation [33]. Kang integrated a Huber-DCS hybrid kernel with the Sage–Husa algorithm to suppress heavy-tailed noise and gross measurement errors [34]. Xu optimized noise updating using an exponentially weighted sliding window, adapting to multi-source mixed-noise scenarios [35]. Lin introduced an inverse Wishart prior constraint on the system covariance to compensate for filter degradation caused by inaccurate prior system noise parameters [36]. Sun et al. employed innovation consistency detection to constrain abnormal measurements, thereby improving the filtering performance in regions affected by measurement outliers [37]. Cai et al. investigated the generation mechanism of gyro thermal-magnetic coupled heading errors and developed an integrated compensation scheme combining ensemble learning with the Sage–Husa adaptive Kalman filter [38]. The proposed method was capable of predicting and compensating such drift errors during GPS outages, and its effectiveness was validated through both land-based and marine experiments. Although these various optimization schemes have advanced noise tracking efficiency, outlier suppression, and numerical stability, most improvements are unable to separate the contributions of measurement noise and dynamic model errors within the innovation sequence, limiting their ability to balance rapid tracking of time-varying noise with suppression of model mismatch errors [39].

To address the above problems, this study proposes a dual-adaptive ASHKF algorithm with model matching check for GNSS/INS Integrated Navigation, hereafter referred to as iSage–Husa. The main contributions of this study are summarized as follows. (1) Within the Sage–Husa adaptive filtering framework, an adaptive window-length mechanism is introduced, and a dual-adaptive structure combining local rapid response through a sliding window with global smoothing recursion through a forgetting factor is constructed. This structure enables rapid tracking and stable estimation of time-varying measurement noise. (2) By introducing a model matching test and an upper-bound detection mechanism for the measurement noise covariance, measurement noise disturbances and model mismatch errors can be distinguished and corrected, thereby suppressing the risks of over-adaptation and filter divergence. (3) Experiments are conducted using vehicle-mounted GNSS/INS field data [40], and comparisons with conventional schemes are performed to verify the advantages of the proposed algorithm in terms of accuracy, stability, and robustness under complex dynamic environments.

The remainder of this paper is organized as follows. Section 2 presents the overall system framework and the principles of the different filtering algorithms. Section 3 describes the multi-scenario comparative experiments and analyzes the results to validate the effectiveness of the proposed method. Finally, Section 4 and Section 5 provide the discussion and conclusions, respectively.

## 2. Methods

A GNSS/INS-integrated navigation system based on an error-state filtering framework is adopted in this study. Information fusion between high-frequency strapdown inertial navigation system (SINS) mechanization and low-frequency GNSS positioning measurements is performed to achieve continuous and reliable positioning and attitude estimation.

A 15-dimensional SINS error-state vector is employed as the core filtering state, including attitude errors, velocity errors, position errors, accelerometer bias errors, and gyroscope bias errors. The propagation and coupling characteristics of these error states are described by the SINS error-state equations, from which the filter prediction model is established. For the measurement model, position and velocity updates are adopted. GNSS-derived position and velocity measurements are utilized as observations, and measurement innovations are formed from the differences between GNSS measurements and the corresponding outputs generated by INS mechanization, thereby completing the filter update process.

**Figure 1:**
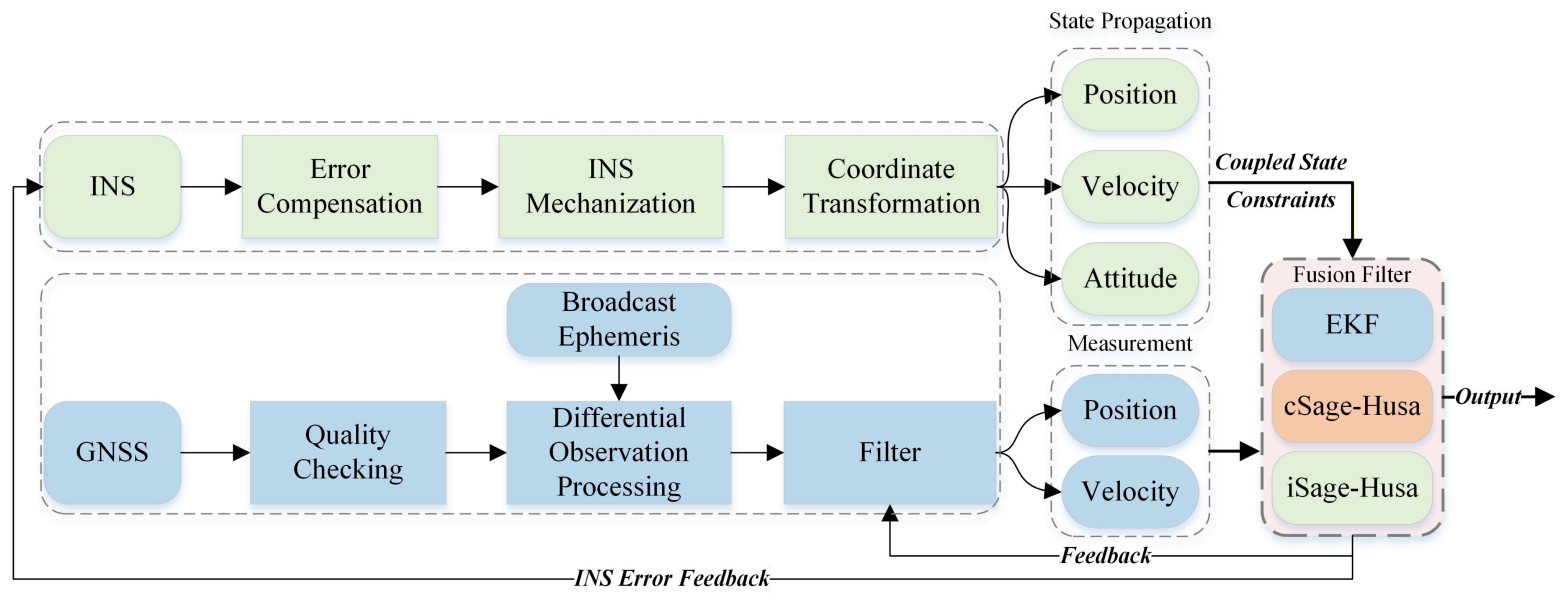
Semi-tightly coupled GNSS/INS architecture.

Within the filtering framework, SINS mechanization is executed at a high frequency to provide real-time predictions of position, velocity, and attitude, whereas GNSS measurements are supplied at a lower update rate to provide absolute positioning information. The accumulated INS errors are estimated and compensated through the filtering process. The estimated error states are fed back to the INS solution in real time to realize closed-loop correction. The corrected GNSS/INS integrated navigation solution is subsequently used as the reference state for the next filtering cycle, thereby improving the continuity, stability, and accuracy of navigation results in complex environments, as shown in Figure 1.

Three filtering schemes, namely the EKF, the cSage–Husa, and the iSage–Husa, are implemented within the GNSS/INS fusion module. Comparative experiments are conducted to evaluate and analyze the positioning and attitude-estimation performance of the three filtering strategies.

### 2.1. System Dynamic Model

**Figure 2:**
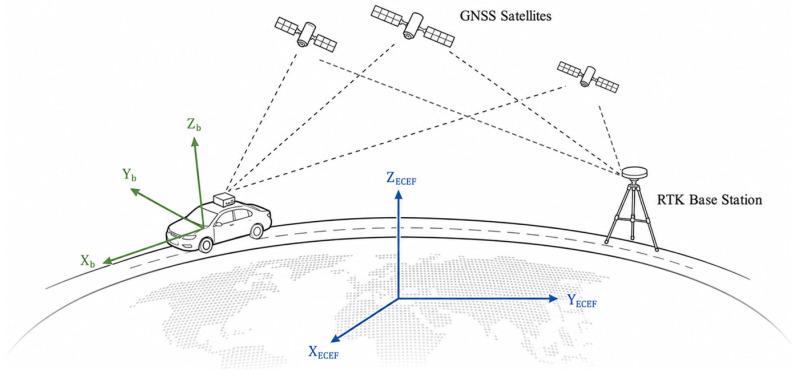
System Model and Coordinate Frames.

The system error-state vector is defined as

(1) $$x_{INS}^{n}={\left[\begin{array}{ccccc} \phi^{n} & \delta v^{n} & \delta p^{n} & \nabla^{b} & \varepsilon^{b} \end{array}\right]}^{T}$$

where the superscripts n and b denote the navigation frame (n-frame) and body frame (b-frame), respectively as shown in Figure 2; ϕ denotes the attitude error vector, δv denotes the velocity error vector, and δp denotes the position error vector; ∇ and ε denote the accelerometer bias vector and gyroscope bias vector, respectively.

Accordingly, the INS error-state dynamic equations can be expressed as:

(2) $${\dot{x}}_{INS}^{n}=Fx_{INS}^{n}+Gw_{INS}$$

where F denotes the state transition matrix, G denotes the process noise distribution matrix, and $w_{INS}$ denotes the process noise vector associated with the inertial navigation system (INS). The explicit forms of F, G, and $w_{INS}$ are given as follows:

(3) $$F=\left[\begin{array}{ccccc} F_{\phi \phi } & F_{\phi v} & F_{\phi p} & -C_{b}^{n} & 0_{3\times 3}\\ F_{v\phi } & F_{vv} & F_{vp} & 0_{3\times 3} & C_{b}^{n}\\ 0_{3\times 3} & F_{pv} & F_{pp} & 0_{3\times 3} & 0_{3\times 3}\\ 0_{3\times 3} & 0_{3\times 3} & 0_{3\times 3} & F_{\nabla^{b}} & 0_{3\times 3}\\ 0_{3\times 3} & 0_{3\times 3} & 0_{3\times 3} & 0_{3\times 3} & F_{\varepsilon^{b}} \end{array}\right]$$

where $C_{b}^{n}$ is the coordinate transformation matrix from the b-frame to the n-frame.

(4) $$G=\left[\begin{array}{cccc} -C_{b}^{n} & 0_{3\times 3} & 0_{3\times 3} & 0_{3\times 3}\\ 0_{3\times 3} & C_{b}^{n} & 0_{3\times 3} & 0_{3\times 3}\\ 0_{3\times 3} & 0_{3\times 3} & 0_{3\times 3} & 0_{3\times 3}\\ 0_{3\times 3} & 0_{3\times 3} & I_{3\times 3} & 0_{3\times 3}\\ 0_{3\times 3} & 0_{3\times 3} & 0_{3\times 3} & I_{3\times 3} \end{array}\right]$$

(5) $$w_{INS}={\left[\begin{array}{cccc} w_{\phi } & w_{g} & w_{\nabla } & w_{\varepsilon } \end{array}\right]}^{T}$$

Based on the aforementioned error propagation model, real-time prediction of navigation errors is performed during INS mechanization, and the predicted filter state at the current epoch is obtained accordingly.

### 2.2. Measurement Model

The RTK positioning mode is adopted in the experiment. GNSS observations are processed using a filtering algorithm, and the resulting estimates are used as observation inputs to the system. When a baseline is formed between the rover station r and the base station b, with simultaneous observations of GNSS satellites i and j, the dual-frequency uncombined RTK positioning observation equation can be expressed as:

(6) $$\Delta \nabla P_{r,b}^{i,j}=\Delta \nabla \rho_{r,b}^{i,j}+\Delta \nabla l_{r,b}^{i,j}+\Delta \nabla T_{r,b}^{i,j}+\Delta \nabla \varepsilon_{P}$$

(7) $$\Delta \nabla \varphi_{r,b}^{i,j}=\Delta \nabla \rho_{r,b}^{i,j}-\Delta \nabla l_{r,b}^{i,j}+\Delta \nabla T_{r,b}^{i,j}-\lambda \Delta \nabla N_{r,b}^{i,j}+\Delta \nabla \varepsilon_{\varphi }$$

where Δ∇ denotes the double-difference operator; P denotes the GNSS pseudorange observation; φ denotes the GNSS carrier-phase observation; ρ denotes the geometric distance between the GNSS satellite and the receiver; l denotes the ionospheric delay error; λ denotes the carrier wavelength corresponding to the signal frequency; N denotes the integer ambiguity; T denotes the tropospheric delay error; and ε denotes the measurement noise.

### 2.3. Semi-Tightly Coupled GNSS/INS Filter

#### 2.3.1. Fusion Filter Based on the EKF

The state prediction is performed using the INS mechanization results and can be expressed as follows:

(8) $$x_{k}^{-}=x_{INS}^{n}$$

The measurement innovation can be expressed as:

(9) $$z_{k}=\left[\begin{array}{c} p_{GNSS}^{n}\\ v_{GNSS}^{n} \end{array}\right]-\left(\left[\begin{array}{c} p_{INS}^{n}\\ v_{INS}^{n} \end{array}\right]+\left[\begin{array}{c} TC_{b}^{n}L^{b}\\ C_{b}^{n}(\omega_{eb}^{n}\times )L^{b} \end{array}\right]\right)$$

where $p_{GNSS}^{n}$ and $p_{INS}^{n}$ denote the GNSS-derived position and the INS-derived position, respectively; $v_{GNSS}^{n}$ and $v_{INS}^{n}$ denote the GNSS-derived velocity and the INS-derived velocity, respectively; T denotes the transformation matrix from the Earth-Centered Earth-Fixed (ECEF) coordinate system to the geodetic coordinate system; $C_{b}^{n}$ denotes the coordinate transformation matrix from the body frame (b-frame) to the navigation frame (n-frame); $L^{b}$ denotes the lever-arm vector; and $\omega_{eb}^{n}$ denotes the skew-symmetric matrix corresponding to the angular velocity of the body frame relative to the Earth frame, expressed in the navigation frame.

When Doppler observations are unavailable, the above equation can be simplified as follows:

(10) $$z_{k}=p_{GNSS}^{n}-\left(p_{INS}^{n}+TC_{b}^{n}L^{b}\right)$$

The Kalman gain is calculated as:

(11) $$K_{k}=P_{k}^{-}H_{k}^{T}{\left(H_{k}P_{k}^{-}H_{k}^{T}+R_{k}\right)}^{-1}$$

where $P_{k}^{-}$ denotes the predicted state covariance matrix, and $R_{k}$ denotes the current measurement noise covariance matrix.

Measurement matrix:

(12) $$H_{k}=\left[\begin{array}{ccccc} 0 & 0 & I_{3\times 3} & 0 & 0\\ 0 & I_{3\times 3} & 0 & 0 & 0 \end{array}\right]$$

Similarly, simplification of the expression is realized in the absence of Doppler observations:

(13) $$H_{k}=\left[\begin{array}{ccccc} 0 & 0 & I_{3\times 3} & 0 & 0 \end{array}\right]$$

Similarly, the above equation can be simplified as follows in the absence of Doppler observations:

(14) $$x_{k}^{+}=x_{k}^{-}+K_{k}z_{k}$$

Posterior covariance:

(15) $$P_{k}^{+}=(I-K_{k}H_{k})P_{k}^{-}$$

Since the posterior covariance matrix of the RTK solution can effectively reflect the current measurement noise level, its diagonal elements are adopted as the measurement noise covariance $R_{k}$ of the fusion filter at the current epoch in the EKF scheme and are subsequently used for integrated navigation filtering.

#### 2.3.2. Fusion Filter Based on cSage–Husa

Based on the unbiased estimation assumption of the EKF, the Gaussian white-noise assumption, the independence between process and measurement noises, and the definition of innovation covariance, the following relationship can be derived:

(16) $$E\left[z_{k}z_{k}^{T}\right]=HP_{k}^{-}H^{T}+R_{k}$$

(17) $$R_{k}=E\left[z_{k}z_{k}^{T}\right]-HP_{k}^{-}H^{T}$$

To estimate the expectation of the innovation covariance, a fading-factor-based recursive formulation is adopted in the conventional Sage–Husa algorithm, which is given by:

(18) $${\hat{C}}_{k}=(1-d_{k}){\hat{C}}_{k-1}+d_{k}z_{k}z_{k}^{T}$$

where

(19) $$d_{k}=\frac{1-b}{1-b^{t}}$$

where b denotes the initial forgetting factor, typically selected within the range of 0.8–0.98, and t represents the time index, which generally corresponds to the current epoch number.

Accordingly, the recursive estimation equation of the measurement noise covariance in the Sage–Husa algorithm can be derived as:

(20) $${\hat{R}}_{k}=(1-d_{k}){\hat{R}}_{k-1}+d_{k}(z_{k}z_{k}^{T}-HP_{k}^{-}H^{T})$$

In practical GNSS/INS integrated navigation applications, however, the above assumptions are rarely satisfied exactly. Especially during high-dynamic carrier motions and GNSS signal degradation or blockage, the innovation sequence contains contributions from both measurement noise and model mismatch errors, which can be expressed as:

(21) $$v_{k}=v_{k}^{noise}+v_{k}^{\mathrm{mod}el}$$

In this case, the conventional cSage–Husa algorithm incorrectly incorporates model errors into the measurement noise estimation, which results in: an abnormal amplification of $R_{k}$; lagged noise estimation; abnormal variations in the filter gain; and sustained error divergence.

Therefore, the core issue of GNSS/INS adaptive filtering does not merely lie in enhancing noise tracking speed, but rather in maintaining the consistency of innovation statistics under conditions of model mismatch.

It is noteworthy that, in practical scenarios, high-dynamic carrier motions can cause the attitude angle estimates to fail to converge in the short term, thereby resulting in abnormal amplification of the innovation sequence. If such disturbances are introduced during the initialization phase of the cSage–Husa noise estimation, the noise estimation bias would be significantly amplified, readily leading to filter divergence or a substantially prolonged convergence time. Conducting comparative analyses based on this scenario is therefore of limited practical significance. Accordingly, the cSage–Husa algorithm described herein implements noise estimation by incorporating the mean of multi-epoch innovation variances on the basis of Equation (17), representing a robust modification better suited to engineering applications [23].

#### 2.3.3. Fusion Filter Based on iSage–Husa

**Figure 3:**
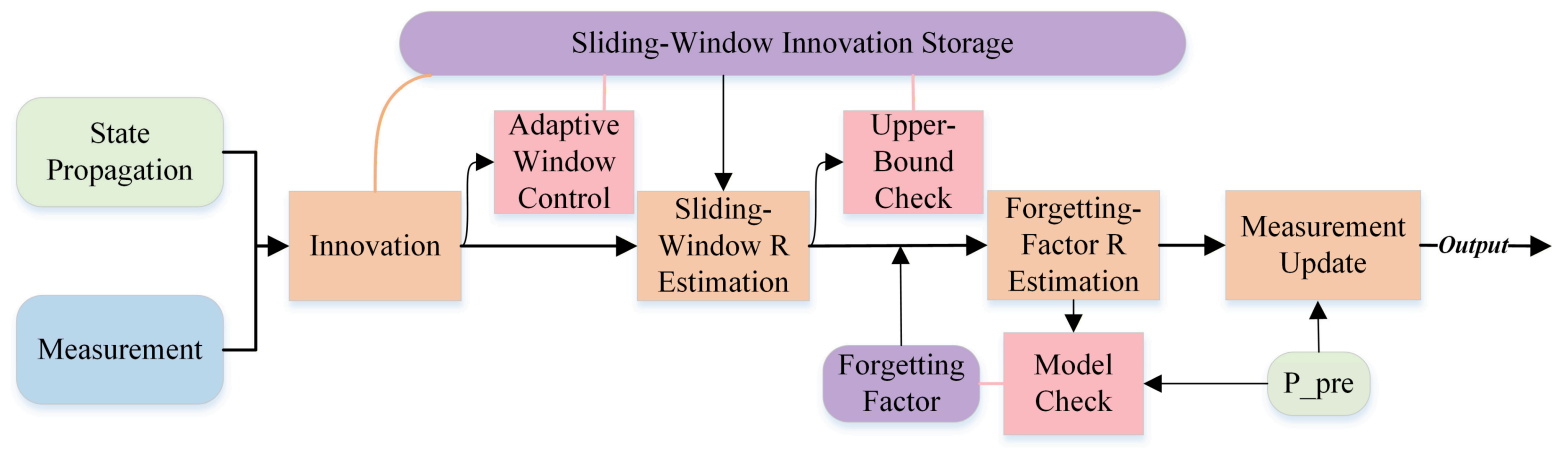
Mechanism of the Dual Adaptive.

To address the convergence difficulty of the cSage–Husa algorithm in GNSS/INS integrated navigation during the initial filtering stage, which is caused by the mismatch between the filter model and the carrier dynamic characteristics, as well as its insufficient tracking response to rapid noise variations in complex scenarios, a window adaptive mechanism is introduced in this study while preserving the temporal recursive property based on the forgetting factor. Considering the requirement of window-based algorithms to balance local rapid response and global smoothing optimality in noise estimation, the weights of historical information are dynamically adjusted through a model-matching test, thereby establishing a dual adaptive mechanism. Consequently, an adaptive filtering method suitable for strong disturbances and rapidly time-varying noise environments is proposed (Figure 3).

(1) The measurement noise covariance matrix estimation equation can be derived based on Equation (17):

(22) $$R_{k}=\frac{1}{N}\sum_{i=k-(N-1)}^{k}z_{i}z_{i}^{T}-HP_{k}^{-}H^{T}$$

where N denotes the current window length.

To quantitatively evaluate the fluctuation characteristics of the innovation sequence, a window-based innovation fluctuation indicator is introduced as follows [33]:

(23) $$\lambda_{k}=\frac{1}{m}\sum_{j=1}^{m}\frac{\left|z_{k}(j)-{\bar{z}}_{k-1}(j)\right|}{{\bar{z}}_{k-1}(j)}$$

where ${\bar{z}}_{k-1}(j)$ denotes the mean absolute value of the j-th innovation component at the previous epoch k−1.

The window scaling calculation formula can then be defined as follows:

(24) $$N_{i+1}=round\left(\frac{N_{i}}{\lambda_{k}}\right)$$

**Figure 4:**
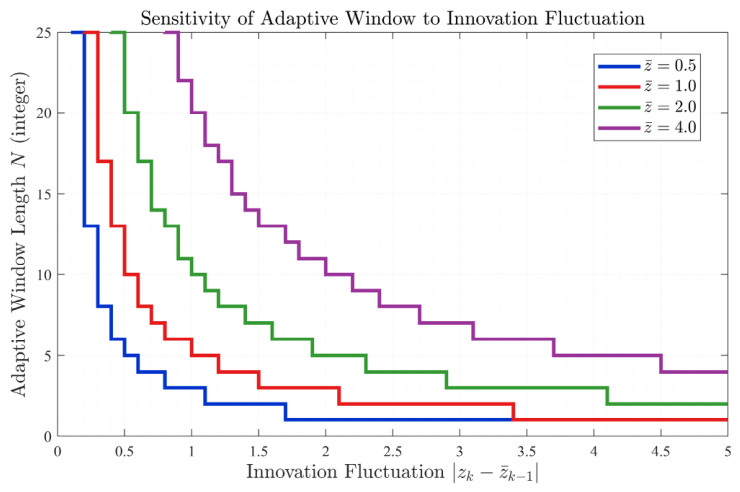
Illustration of the Window Adaptive Function Sensitivity (with an initial window size of 5).

As illustrated in Figure 4, when the observations are stable, the difference $z_{k}(j)-{\bar{z}}_{k-1}(j)$ is significantly reduced, resulting in an appropriately enlarged window; conversely, the window is reduced. To balance the initial statistical stability of the innovation sequence and the tracking capability for time-varying noise, the initial length of the adaptive window is set to 5.

(2) In practical GNSS/INS-integrated navigation applications, complex observation environments readily induce prolonged noise disturbances, resulting in severe fluctuations of the innovation sequence and causing the adaptive sliding window to be persistently compressed within a small range of 1–5 epochs. Such a small window sample size is insufficient to approximate the theoretical mathematical expectation of the innovation covariance through mean statistics, leading to a substantial reduction in the estimation accuracy of the measurement noise covariance R. To address this, a fading factor is introduced to perform weighted recursive updates, which enhances the real-time accuracy of noise estimation while ensuring the overall stability of the filtering process.

The estimation formula for the final measurement noise covariance can be expressed as follows:

(25) $${\hat{R}}_{k}=(1-d_{k}){\hat{R}}_{k-1}+d_{k}R_{k}$$

where $d_{k}$ is calculated according to Equation (19).

**Figure 5:**
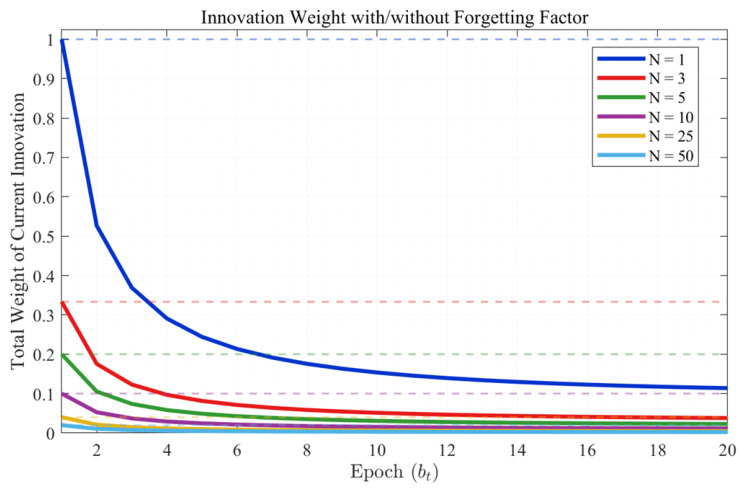
Influence Weight of the Current–Epoch Innovation on Innovation Covariance estimation. (The dashed horizontal lines denote the weight values without the forgetting factor; curves with different colors correspond to different sliding window sizes N.).

Figure 5 presents the evolution of innovation weights over time under different window lengths. Unlike the uniform weighting strategy adopted in conventional fixed-window approaches, the introduction of the forgetting factor enables the innovation weights to exhibit an exponential decay characteristic over time, with smaller window lengths resulting in faster weight attenuation. This mechanism dynamically adjusts the contribution ratio between historical information and current measurements according to the window scale, thereby improving the responsiveness to time-varying noise while maintaining the stability of noise estimation. Meanwhile, it effectively prevents abnormal adjustments of the measurement noise covariance caused by severe innovation fluctuations, thus enhancing filtering stability.

To reduce the influence of model mismatch on innovation statistics under high-dynamic motion conditions, an upper-bound detection module and a model matching test module are introduced after the first-step and second-step measurement noise covariance estimations, respectively. The former is designed to prevent overestimation of the measurement noise covariance, while the latter mitigates covariance underestimation caused by excessive adaptation.

(3) Since GNSS observations are preprocessed and possess relatively high measurement reliability, the variation range of the measurement noise covariance can be constrained by a theoretically determined maximum noise covariance.

For the position measurements, the measurement noise covariance matrix estimated from the innovation sequence within the window is expressed as follows:

(26) $$R_{k}=\left[\begin{array}{ccc} {\hat{r}}_{E,k} & 0 & 0\\ 0 & {\hat{r}}_{N,k} & 0\\ 0 & 0 & {\hat{r}}_{U,k} \end{array}\right]$$

where ${\hat{r}}_{E,k}$, ${\hat{r}}_{N,k}$, and ${\hat{r}}_{U,k}$ denote the estimated measurement noise variances in the east, north, and up channels at the current epoch, respectively.

The theoretical upper-bound matrix can be expressed as follows:

(27) $$U=\left[\begin{array}{ccc} u_{E} & 0 & 0\\ 0 & u_{N} & 0\\ 0 & 0 & u_{U} \end{array}\right]$$

where the subscripts E, N, and U denote the east, north, and up directions, respectively, and $u_{i}$ represents the theoretical maximum measurement noise variance in the corresponding direction, which is determined according to the positioning mode, prior sensor accuracy specifications, and observation environment.

The normalized noise constraint statistic is defined as follows:

(28) $$\eta_{k}=\max\left(\begin{array}{ccc} \frac{{\hat{r}}_{E,k}}{u_{E}}, & \frac{{\hat{r}}_{N,k}}{u_{N}}, & \frac{{\hat{r}}_{U,k}}{u_{U}} \end{array}\right)$$

Accordingly, a covariance correction update rule is constructed for cases where the measurement noise covariance exceeds the upper bound:

(29) $$\left\{\begin{array}{cc} if & \eta_{k}\geq 1\\ N_{k+1}=1, & {\hat{R}}_{k+1}=diag(P_{GNSS,k+1}^{+}) \end{array}\right.$$

(4) When the measurement noise covariance is underestimated, directly adopting the theoretical highest accuracy as the lower bound of the covariance is inappropriate due to the strong dependence of GNSS measurement accuracy on the observation environment. Therefore, a model matching test is introduced to constrain the measurement noise covariance estimate obtained after incorporating the forgetting factor.

The statistic is defined as follows:

(30) $$\gamma_{k}=v_{k}^{T}S_{k}^{-1}v_{k}$$

where:

(31) $$S=HP_{k}^{-}H^{T}+{\hat{R}}_{k}$$

S denotes the theoretical innovation covariance.

To judge whether the current innovation sequence satisfies the theoretical statistical characteristics, the chi-square test is adopted for model matching detection, and a threshold can be set as follows:

(32) $$T=\chi_{a}^{2}(m)$$

If $\gamma_{k}>T$, the current innovation value is judged to be statistically mismatched. At this time, the forgetting factor is adjusted to rapidly reduce the weight of historical statistical information, thereby improving the filter’s tracking performance against abrupt noise, as expressed below:

(33) $$t_{k+1}=t_{a}$$

**Figure 6:**
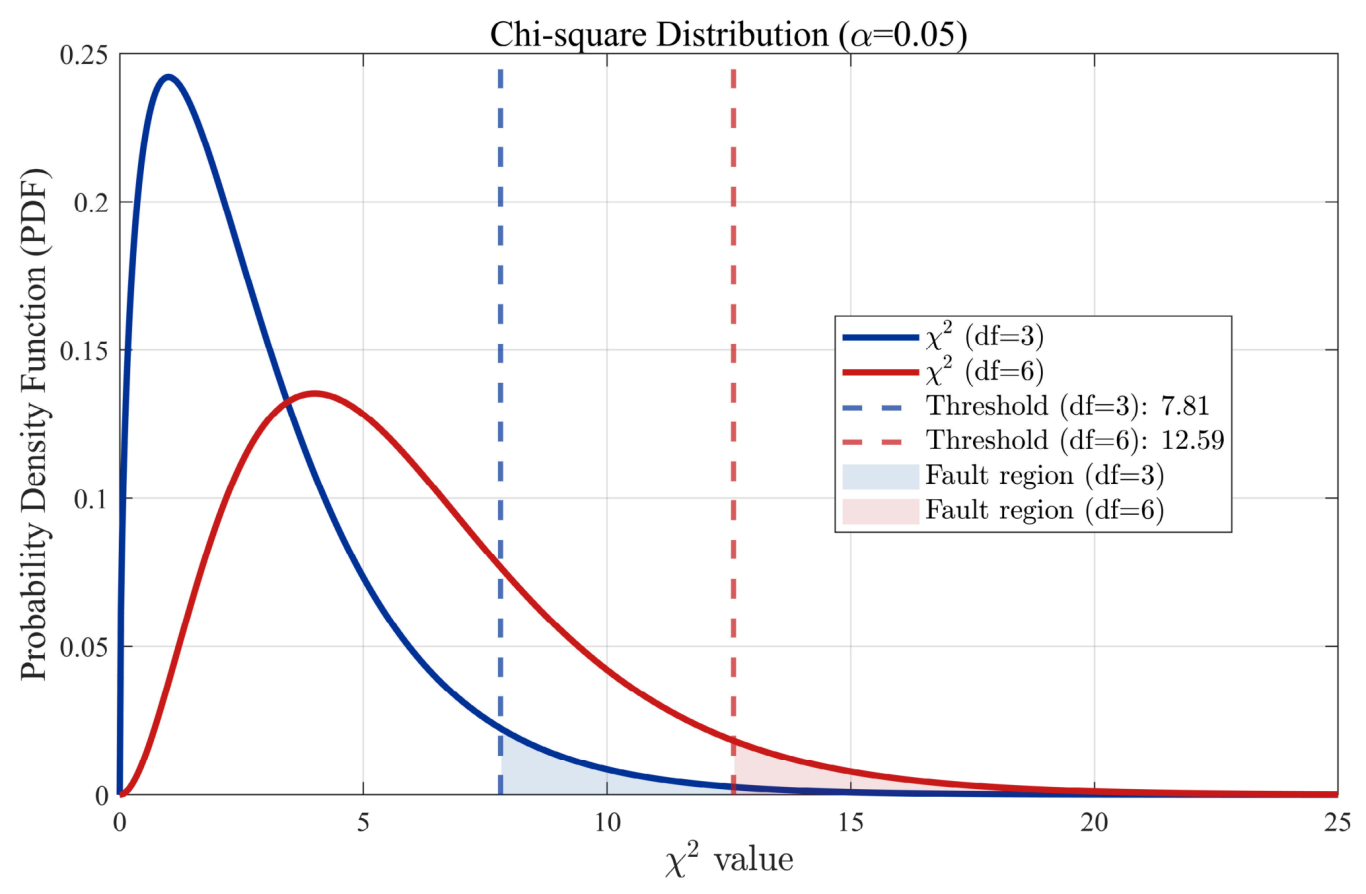
Chi-Square Probability Distribution.

The matching test threshold T=7.81 is derived from the chi-square distribution with 95% confidence and 3 degrees of freedom. The tunable parameter $t_{a}$ varies with the observation environment and is assigned a value of 10 in this study, as shown in Figure 6.

## 3. Experimental Analysis

### 3.1. Experimental Data

The open-source vehicle-borne GNSS/INS field test dataset is adopted in the experiments. Collected and publicly released in 2025, this dataset is available for performance evaluation of integrated navigation algorithms.

The dataset consists of raw GNSS observations, IMU measurements and reference ground truth trajectories.

Two sets of IMU devices are utilized in the experiments. Among them, ADIS16470 (Analog Devices, Inc., Wilmington, MA, USA), a low-cost navigation-grade IMU, is employed for the validation of the integrated navigation algorithm; StarNeto XW-GI7660 (StarNeto, Beijing, China), a high-precision tactical-grade IMU, is used to compute the reference trajectory. The instrument parameters are listed in Table 1.

**Figure 7:**
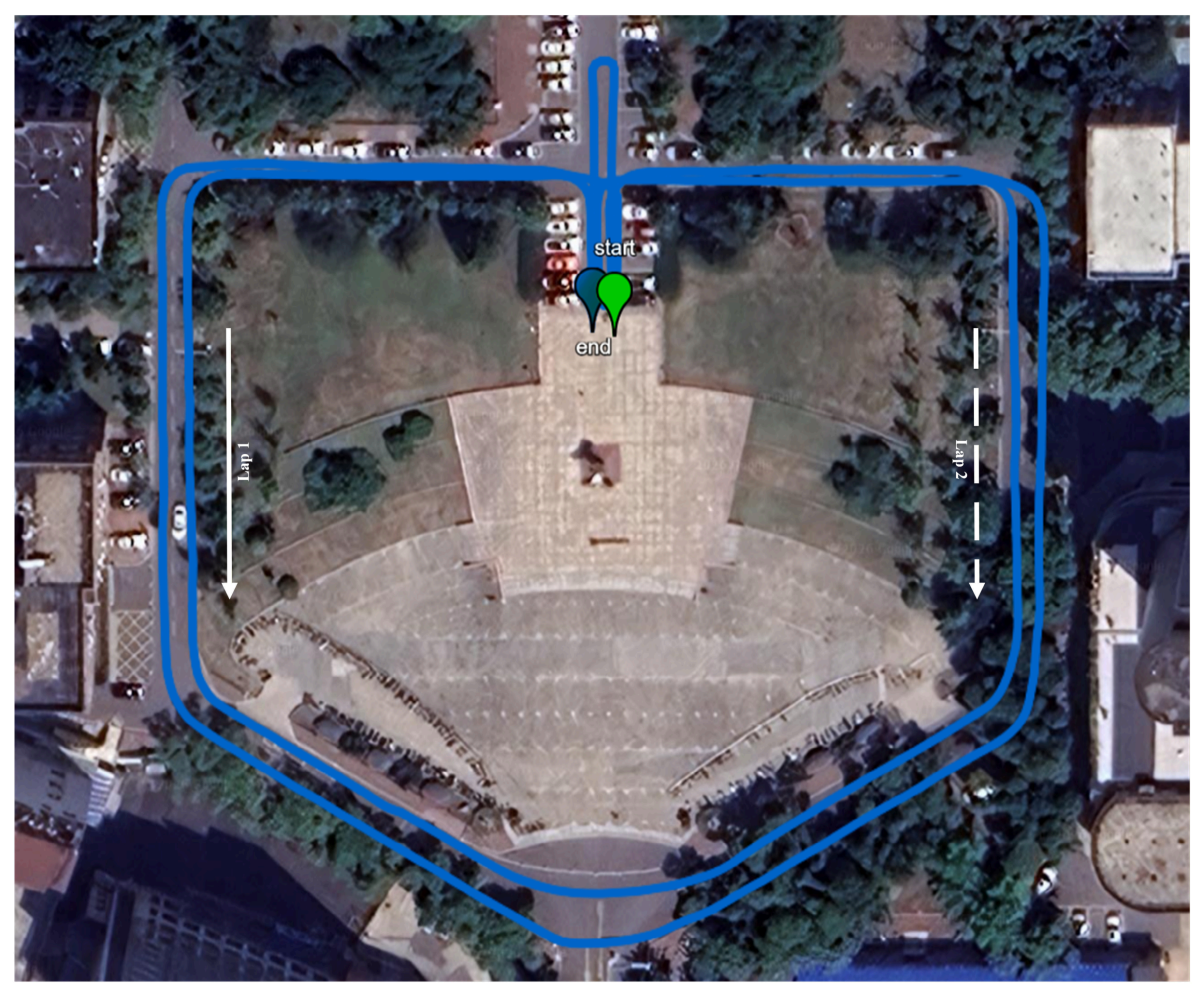
Experimental Route and Scenarios.

Three typical complex scenarios are covered in the experimental setup, namely instantaneous GNSS interference, continuous weak GNSS observation, and high-dynamic-motion environments, as shown in Figure 7.

To evaluate algorithm performance, comparisons are made among: EKF; cSage–Husa; and the proposed algorithm (iSage–Husa).

### 3.2. Performance Evaluation of the Proposed Algorithm

#### 3.2.1. Algorithm Performance Analysis Under Large-Angle Maneuvers and Instantaneous GNSS Interference During the Initialization Phase

This section selects a complete experimental scenario involving the initial high-dynamic motion of the carrier and instantaneous GNSS interference to validate the algorithm performance under complex dynamic conditions.

**Figure 8:**
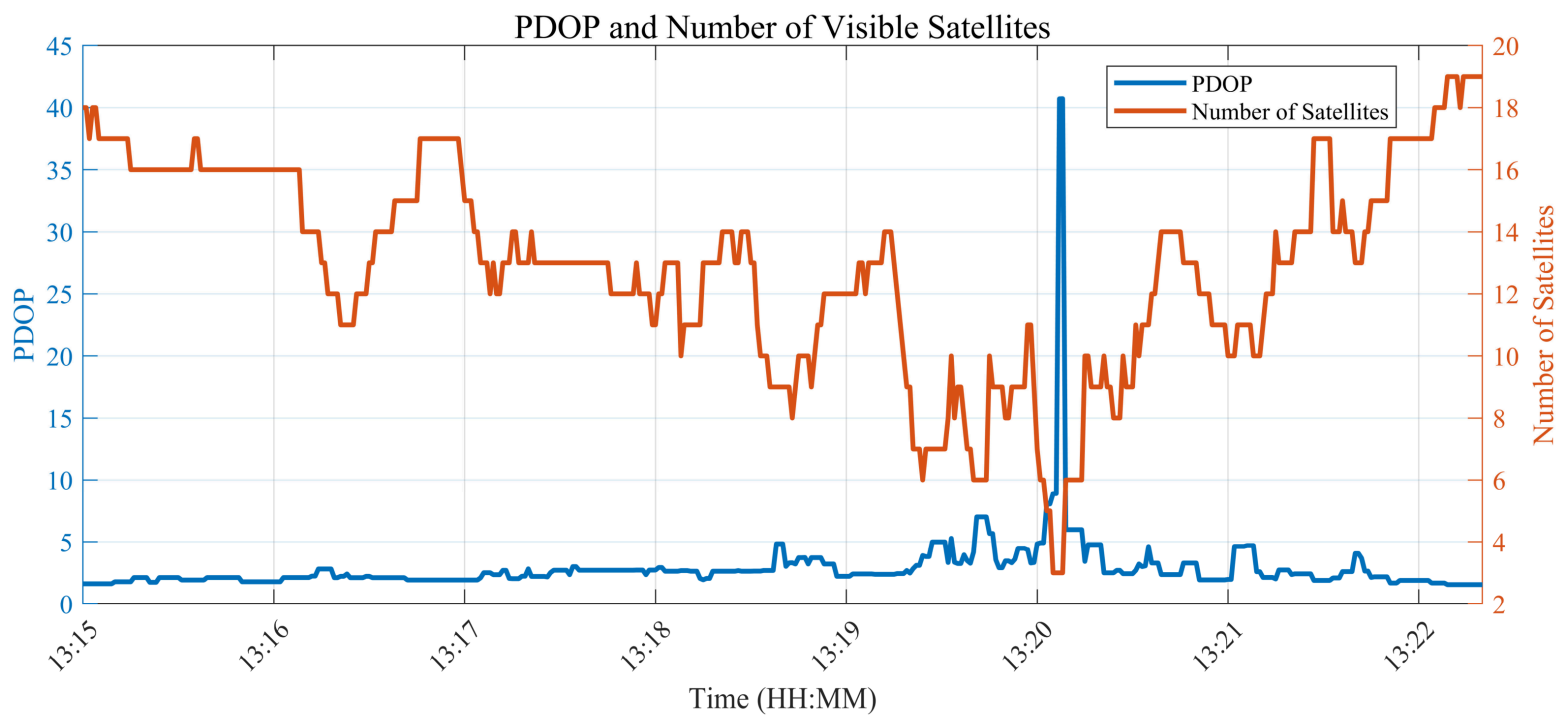
PDOP and Number of Available Satellites in Experiment 1.

Figure 8 presents the GNSS observation quality statistics for the entire experiment. The variations in the number of available satellites and PDOP values clearly reflect the dynamic fluctuations of the observation conditions. Specifically, the number of available satellites varies drastically between 2 and 18, and the PDOP exhibits a pronounced peak around 13:20, corresponding to an instantaneous interference scenario with severely degraded GNSS geometric configuration.

**Figure 9:**
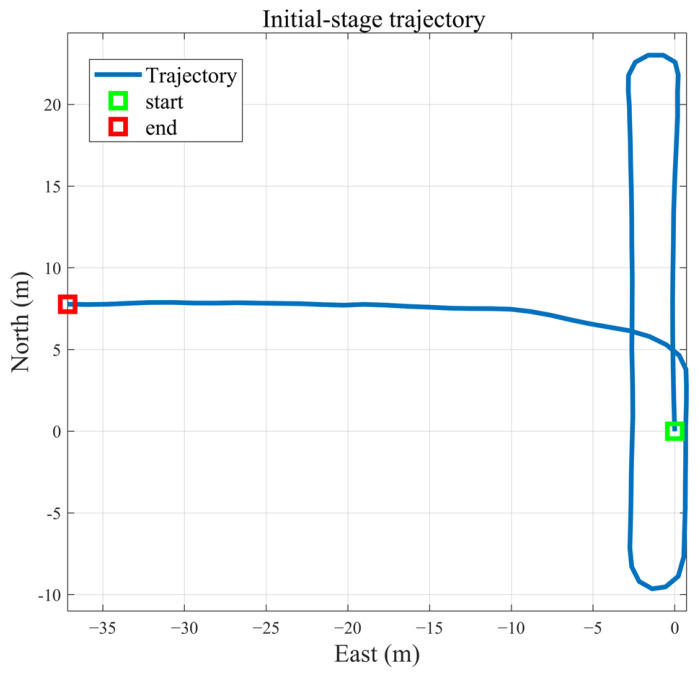
Trajectory Illustration during the Initialization Phase in Experiment 1.

Figure 9 presents a close-up of the carrier’s motion trajectory during the initialization phase under the experimental scenario. In the early stage, the carrier accelerates along a straight path in the horizontal direction, while in the later stage, it undergoes high-dynamic motions involving multiple abrupt stops and turns, resulting in significant attitude changes. This motion pattern introduces substantial model errors, which, when coupled with the time-varying noise effects caused by subsequent GNSS observation degradation, impose dual challenges on the filter’s model matching and noise adaptation capabilities.

This experimental scenario comprehensively covers the typical characteristics of carrier motion model errors and time-varying GNSS measurement noise, thereby enabling an effective evaluation of the robustness and convergence performance of different algorithms under complex environments.

**Figure 10:**
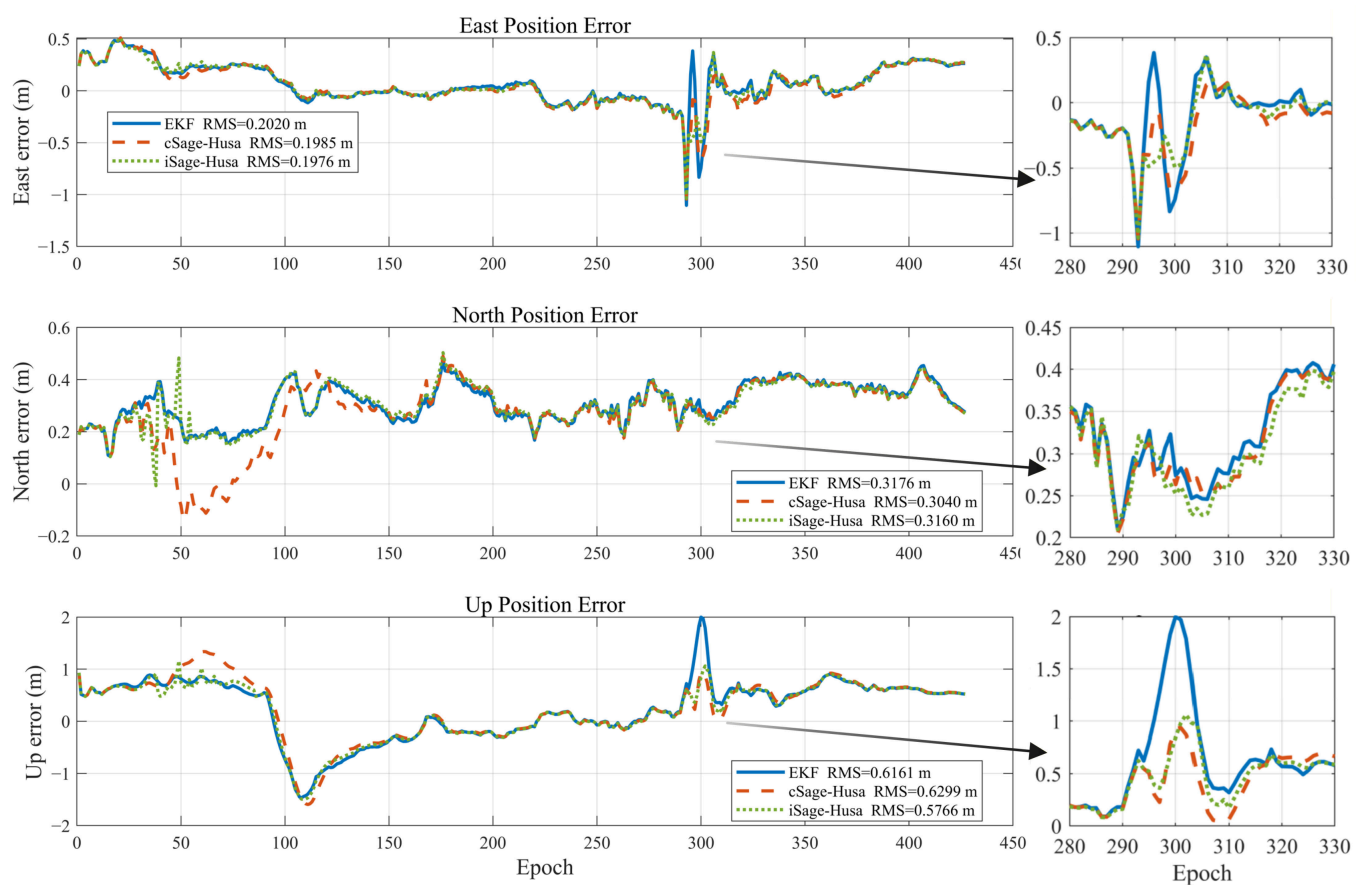
Position Error Analysis in Experiment 1.

**Figure 11:**
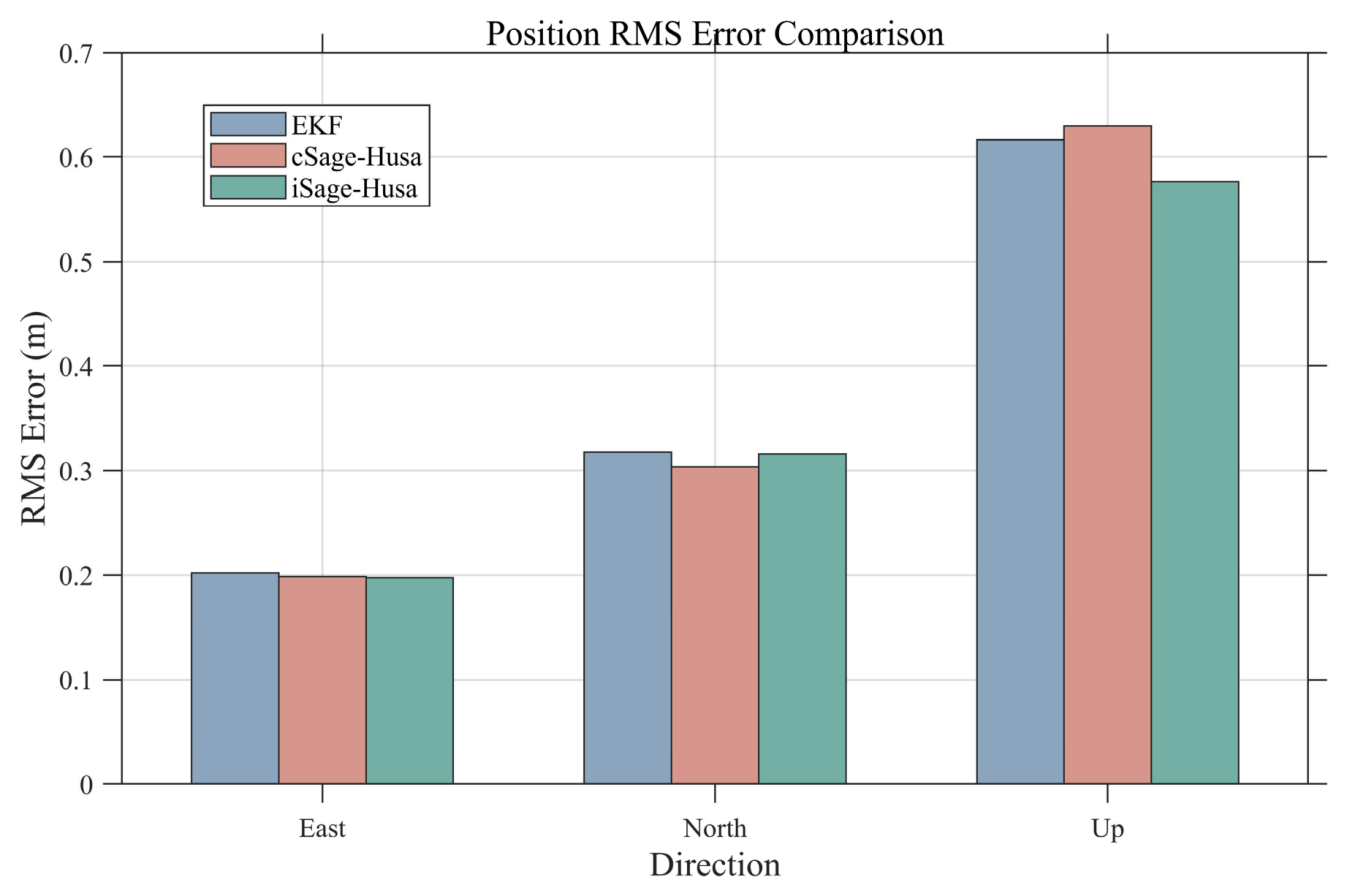
Overall Position RMS Statistics in Experiment 1.

(1) As shown in Figure 10 and Figure 11, from the overall results, the conventional EKF algorithm exhibits severe error fluctuations during the strong-denial peak periods, with the peak error in the up channel approaching 2 m, indicating insufficient robustness against GNSS observation interruptions and dynamic motion. Although the cSage–Husa algorithm slightly improves the horizontal errors, its up-channel error is further amplified compared with that of the EKF, demonstrating insufficient adaptability to scenarios involving the coupling of strong dynamics and severe interference. In contrast, the improved algorithm proposed in this study exhibits superior stability and robustness in the east, north, and up channels. Specifically, the RMS error in the up channel is reduced by approximately 6.4% compared with that of the EKF and by approximately 8.5% compared with that of the cSage–Husa algorithm. Meanwhile, the error fluctuations in the east channel and north channel components are also effectively suppressed, and the overall accuracy is superior to that of the cSage–Husa algorithm. In particular, during the periods in which denial peaks and high-dynamic carrier motions coincide are superimposed, the improved algorithm effectively suppresses error divergence, demonstrating strong disturbance rejection capability under extreme GNSS observation environments and high-dynamic-motion scenarios. Thus, significant improvements in positioning accuracy and robustness are achieved under severe interference conditions.

As indicated by the standard deviation (STD) statistics in Table 2, the proposed improved algorithm achieves higher positioning accuracy than the cSage–Husa algorithm in the east, north, and up directions, with stronger overall stability. Among these components, the improvement in the north direction is the most significant, with the RMS error decreasing from 0.1236 m to 0.0792 m, corresponding to a relative reduction of 35.9%. This indicates that the window-based adaptive noise estimation effectively enhances the tracking capability of the algorithm for dynamic noise. Moreover, after the introduction of the forgetting factor, the limitation of the pure window-based method, which excessively pursues local optimality while neglecting global stationarity, is further compensated. Consequently, a balance between local noise tracking and global estimation stability is achieved, resulting in more reasonable noise estimation and a more stable filtering process.

**Figure 12:**
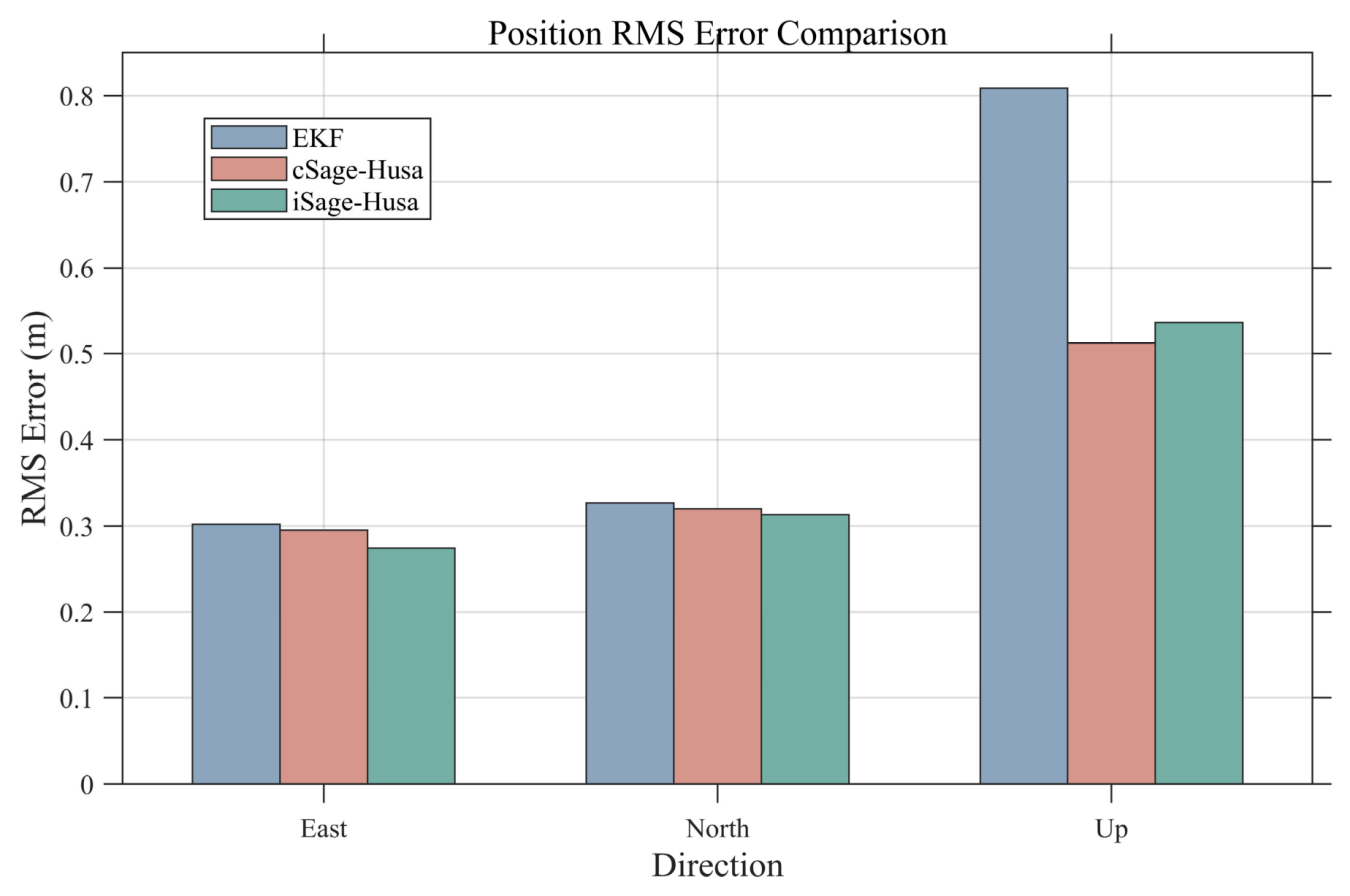
Position RMS Statistics during the Instantaneous Interference Interval in Experiment 1.

A further analysis was conducted on the GNSS-interfered local period. As shown in Figure 12, the differences in position RMS errors among the three algorithms are more pronounced. In the east channel, the RMS errors of EKF, cSage–Husa, and iSage–Husa are 0.3022 m, 0.2950 m, and 0.2739 m, respectively, with iSage–Husa reducing the error by approximately 9.4% compared with EKF and 7.2% compared with cSage–Husa. In the north channel, the corresponding errors are 0.3266 m, 0.3195 m, and 0.3130 m, with iSage–Husa achieving reductions of approximately 4.2% and 2.0% relative to EKF and cSage–Husa, respectively. In the up channel, the EKF error reaches 0.8087 m, whereas the cSage–Husa and iSage–Husa errors are reduced to 0.5122 m and 0.5363 m, representing reductions of 36.7% and 33.7%, respectively.

In the horizontal plane, the errors of the three algorithms remain at similar levels, indicating limited improvement in horizontal positioning accuracy from adaptive noise estimation. The up-channel error differences arise from the accumulation of INS propagation errors following GNSS outages; EKF fails to rapidly converge after GNSS signal recovery, leading to sustained elevated errors. In contrast, the two adaptive algorithms, through real-time estimation of the measurement noise covariance R, effectively suppress up-channel error accumulation, achieving positioning accuracy significantly superior to that of EKF.

**Figure 13:**
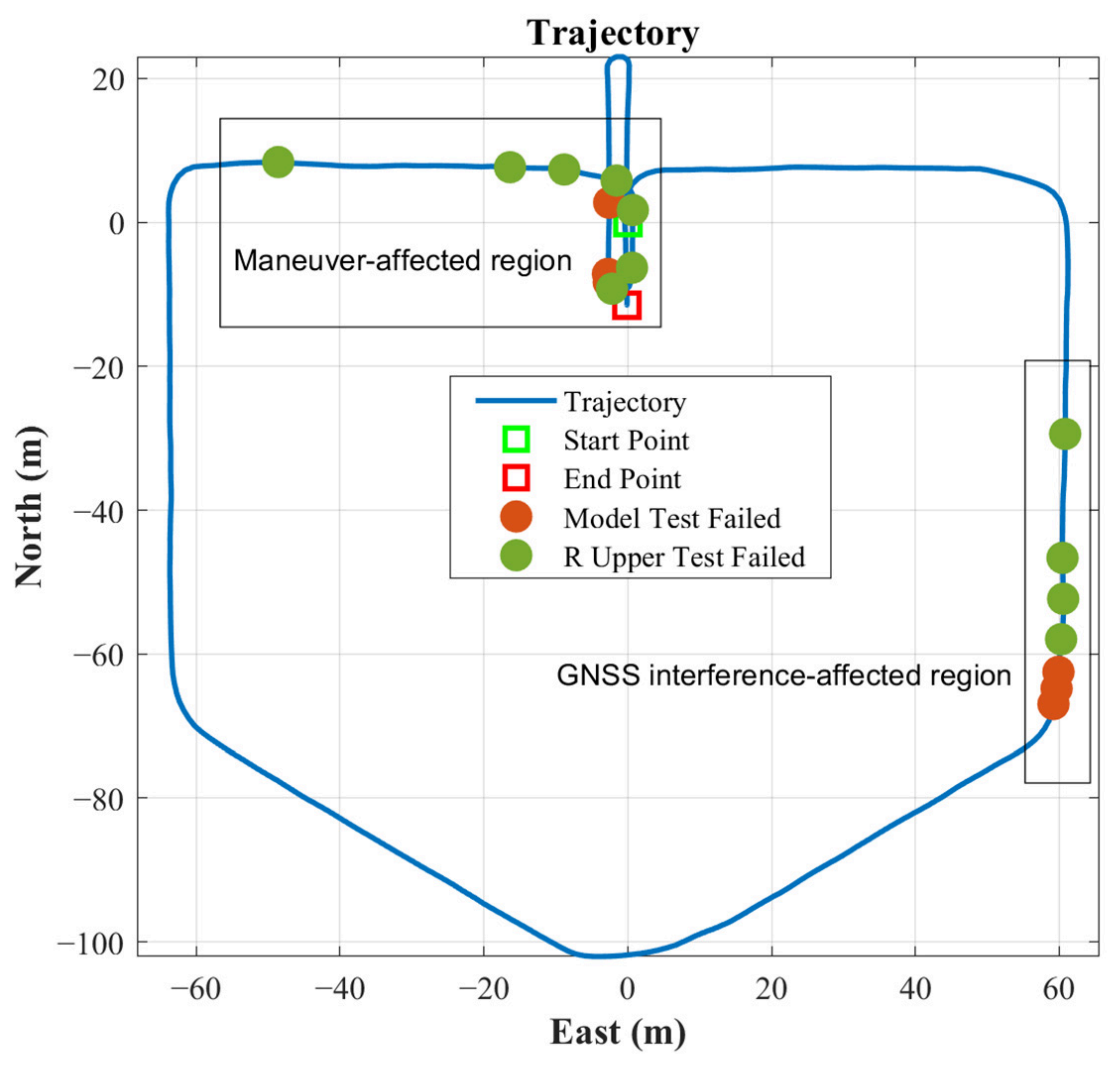
Out-of-Threshold Epochs Identified by the Model Matching Test in Experiment 1.

(2) Figure 13 records the epochs determined to exceed the preset thresholds by the model matching test and the upper-bound test module for (R), together with their spatial distribution. As can be observed from the figure, the out-of-threshold epochs are accurately concentrated in the high-maneuver regions of the carrier and the GNSS-interfered regions, which is consistent with the actual experimental conditions.

In the GNSS-interfered region, when the vehicle enters a road section with vegetation occlusion, the GNSS signal quality deteriorates and the observation residuals increase significantly, thereby triggering the model matching tester. In this case, the filter enhances its assimilation capability for current information by adjusting the forgetting factor, enabling effective estimation of time-varying measurement noise.

When the vehicle leaves the occluded region and the GNSS observation conditions gradually recover, the system has relied only on INS-based position and attitude propagation during the GNSS-denied interval, which introduces significant model bias. This bias is captured by the upper-bound detector for (R). Subsequently, the filter resets the historical innovation window and reinitializes the measurement noise estimation based on the diagonal matrix of the GNSS posterior covariance, and this mechanism directly contributes to the improvement of positioning accuracy in the east channel and north channel.

**Figure 14:**
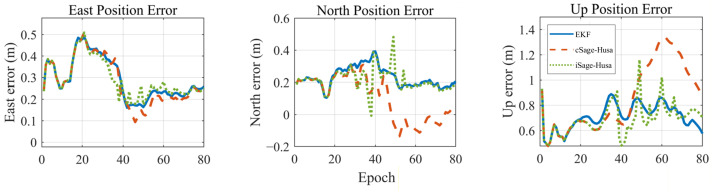
Comparison of Algorithm Position Errors during the Initialization Phase in Experiment 1.

During the initialization phase, the carrier undergoes high-dynamic motions before filter convergence is achieved. As shown in Figure 14, the forgetting factor of the cSage–Husa algorithm remains at a relatively low level during this period, resulting in an excessively high absorption weight assigned to the innovations by the filter. This leads to a severe bias in noise estimation in the up channel and causes rapid error accumulation. Owing to the aforementioned robust modification, the positioning accuracy of the cSage–Husa algorithm in the east channel is comparable to that of the other two algorithms, and its accuracy in the northward direction is even superior to those of the other two algorithms.

**Figure 15:**
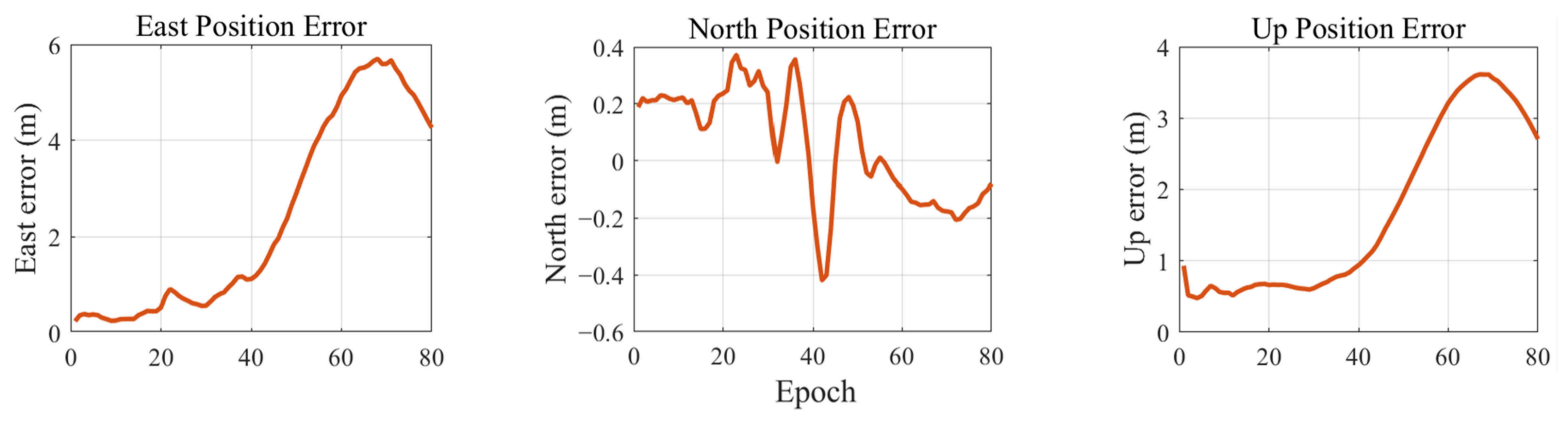
Original Sage–Husa Algorithm without Robust Processing in Experiment 1.

To further illustrate the necessity of this approach, the results of the original Sage–Husa algorithm without robust processing are additionally presented in Figure 15. It can be observed that, in this region, the errors of the original algorithm are an order of magnitude higher than those of the other two algorithms. The positioning results are completely invalid and therefore do not provide a meaningful reference for comparison.

For the proposed algorithm, as shown in Figure 13, the abnormal amplification of innovations caused by filter non-convergence is identified by the model matching test module. The algorithm then rapidly adjusts the measurement noise estimation by modifying the forgetting factor. Subsequently, the anomalous state of this noise estimation is captured by the upper-bound test module for R, prompting the filter to reset the historical innovation window and reinitialize the noise estimation based on the diagonal matrix of the GNSS-derived posterior covariance. After multiple rounds of dynamic adjustment, the filter ultimately achieves stable convergence, maintaining positioning accuracy comparable to that of the EKF during this phase.

**Figure 16:**
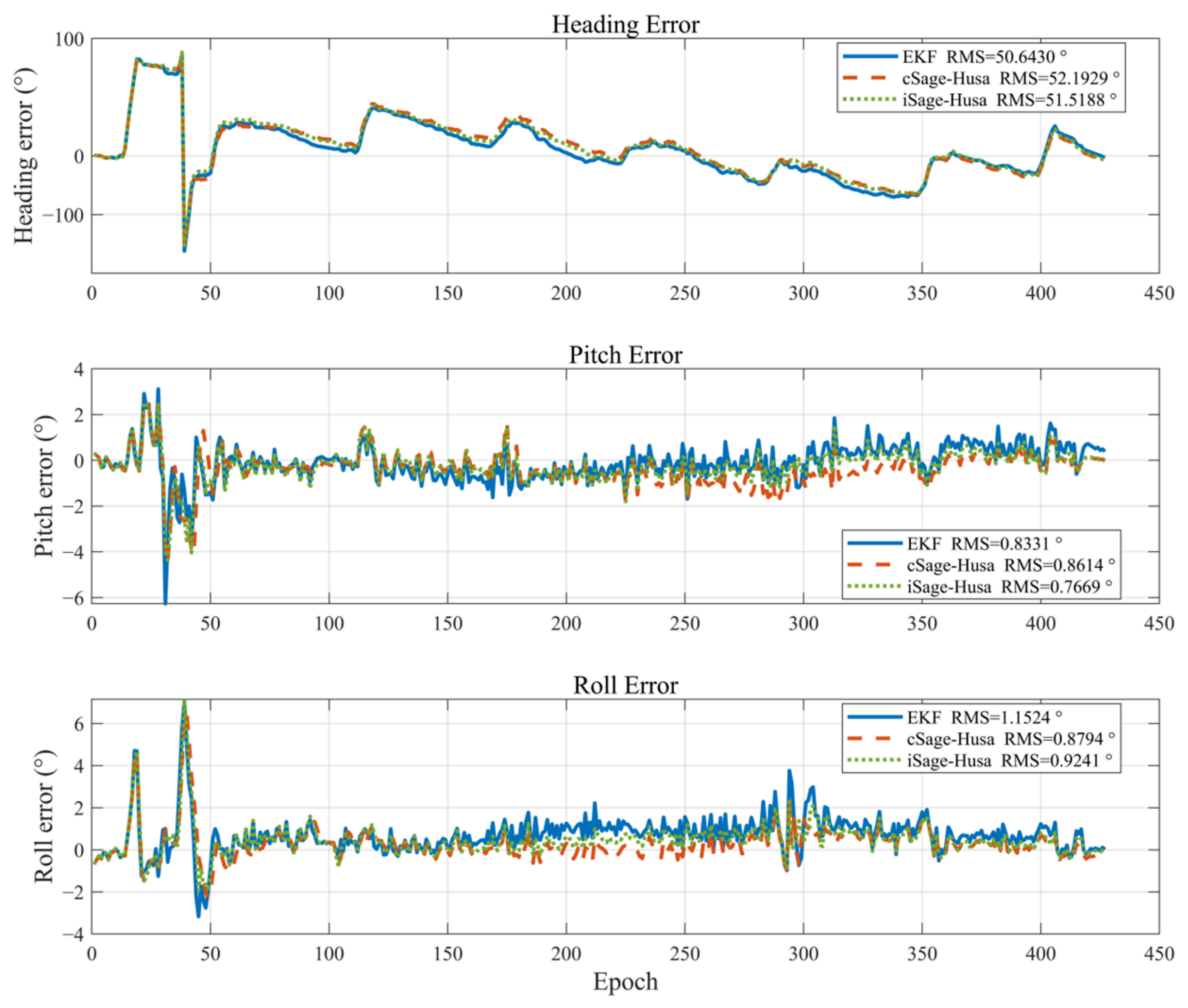
Attitude Error Analysis in Experiment 1.

(3) In terms of attitude estimation, as shown in Figure 16, the heading angle exhibits short-term fluctuations due to high-dynamic carrier motions involving repeated turns during the initialization phase, followed by rapid convergence and stabilization. The pitch and roll angles are more strongly constrained by the intrinsic observations of the IMU, resulting in smaller overall fluctuations and faster convergence. This indicates that the horizontal attitude is naturally insensitive to GNSS observation interruptions and external environmental disturbances.

A comparison of the three methods shows that, in terms of heading estimation, the performance differences among the conventional EKF, cSage–Husa, and iSage–Husa algorithms are relatively small. This is mainly because GNSS observations are affected only during local intervals in this environment, and all three filters can achieve sufficient convergence, resulting in similar overall performance. For the pitch and roll components, the two Sage–Husa adaptive filters exhibit better noise suppression capability than the EKF. In particular, the RMS errors of the proposed iSage–Husa algorithm are 0.7669° and 0.9241°, respectively, effectively alleviating the performance degradation of the conventional cSage–Husa algorithm in this scenario. Overall, even under complex conditions characterized by severe interference and strong dependence on INS propagation, the proposed improved algorithm can achieve higher horizontal attitude estimation accuracy through optimized measurement noise estimation and a motion-state adaptation mechanism.

#### 3.2.2. Algorithm Performance Analysis Under Continuous GNSS Interference

This section selects an experimental scenario characterized by continuous weak GNSS observations and repeated PDOP fluctuations to validate the performance of the algorithm under long-term low-quality observation conditions.

**Figure 17:**
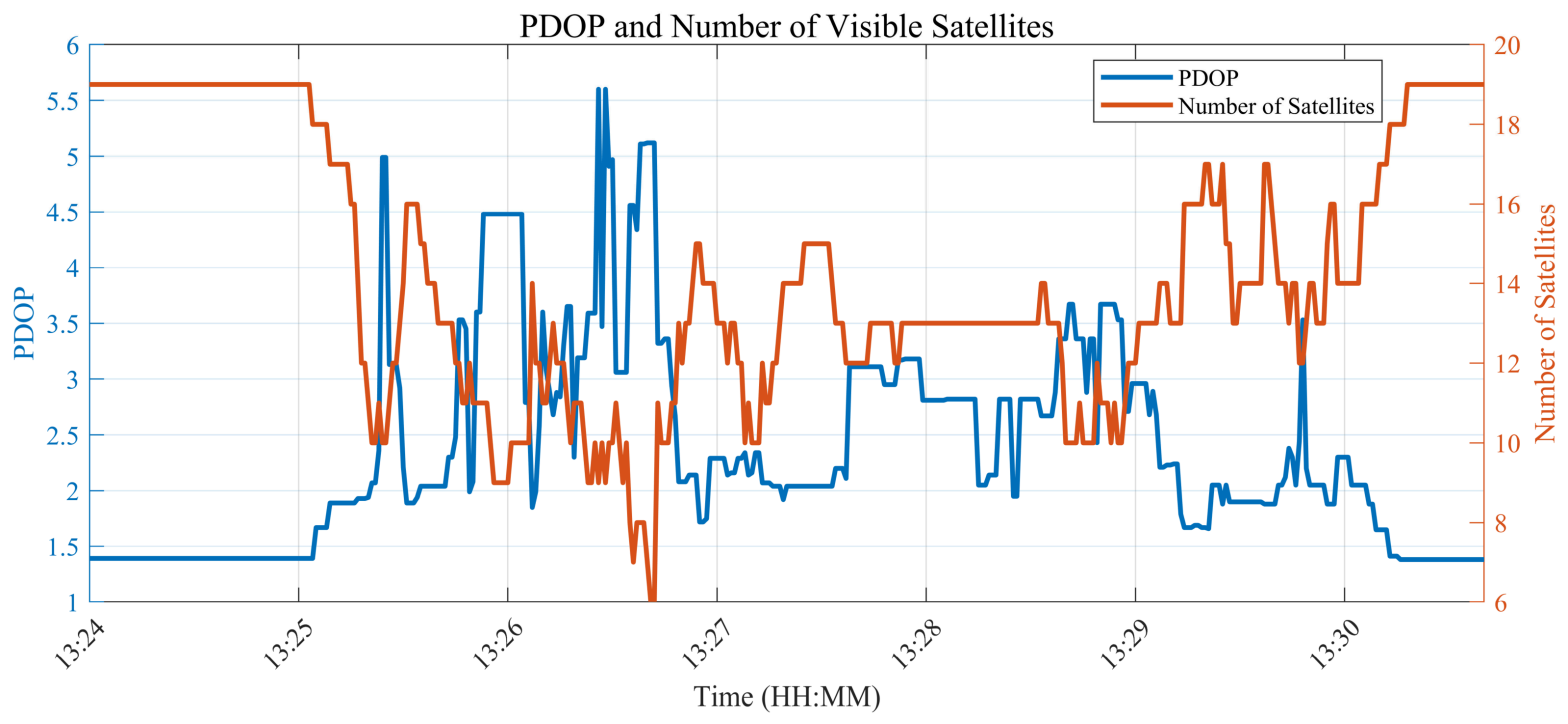
PDOP and Number of Available Satellites in Experiment 2.

Figure 17 presents the GNSS observation quality statistics during this period. The variations in the number of available satellites and PDOP values clearly reflect the sustained degradation of observation conditions. Specifically, the number of available satellites gradually decreases from the initial 19 to fluctuate between 6 and 10, while the PDOP rises from approximately 1.5 to remain at elevated levels of 2.0–5.5 during 13:25–13:30, with occasional spike fluctuations corresponding to a disturbance scenario in which the GNSS geometric configuration continuously deteriorates.

**Figure 18:**
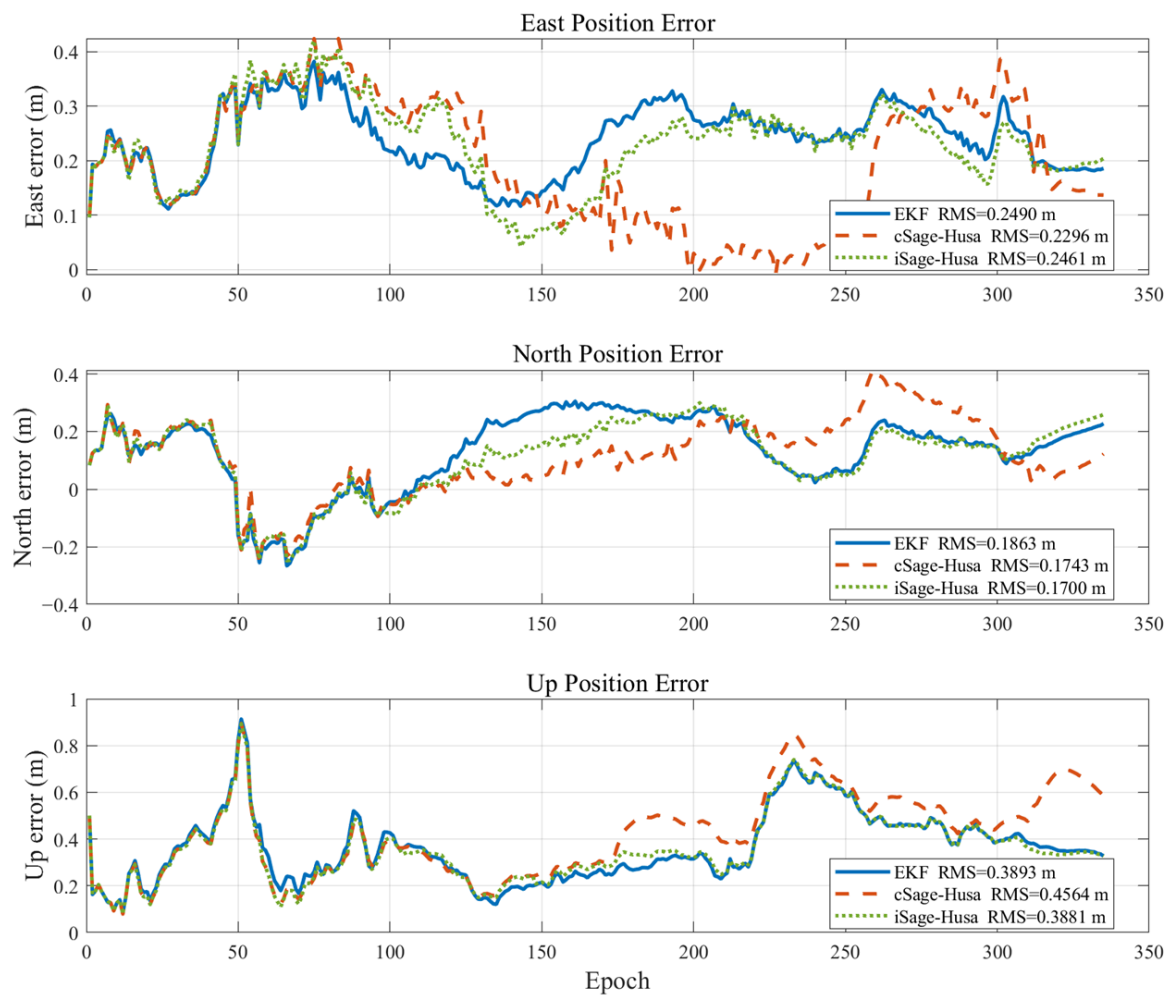
Position Error Analysis in Experiment 2.

**Figure 19:**
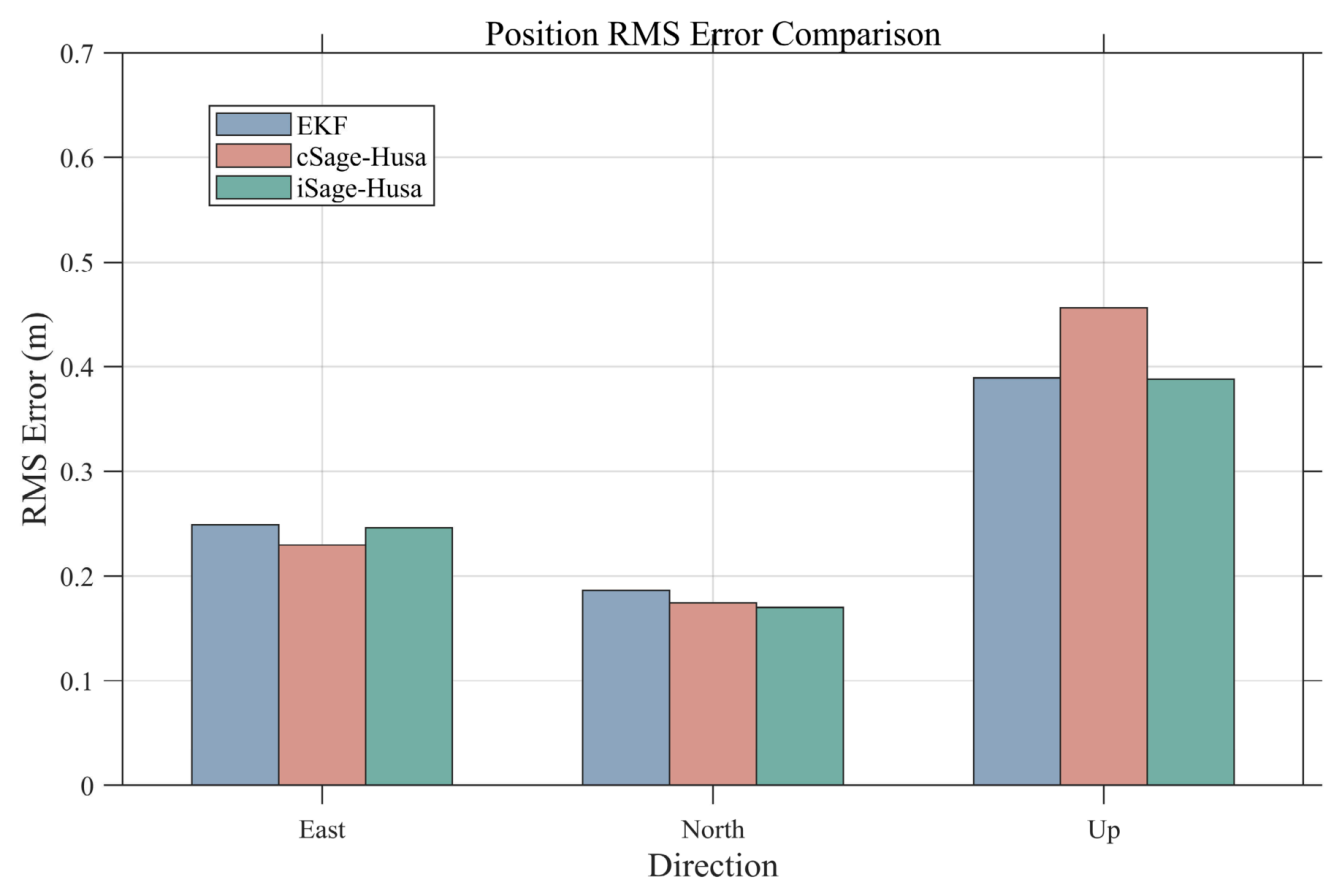
Overall Position RMS Statistics in Experiment 2.

(1) As shown in Figure 18 and Figure 19, the proposed improved algorithm reduces the northward error by approximately 8.7% compared with the EKF. Compared with the cSage–Husa algorithm, the northward error is reduced by approximately 2.5%, the up-channel error is reduced by approximately 15.2%, and the east channel error remains essentially equivalent, effectively avoiding the over-adaptation issue and achieving a balance between positioning accuracy and stability under continuous GNSS interference.

The standard deviation statistics in Table 3 reflect the stability of the algorithms. Compared with the cSage–Husa algorithm, the proposed improved algorithm exhibits a clear suppression of error fluctuations in the east, north, and up channels, with significant overall stability enhancement. Specifically, the east channel standard deviation decreases from 0.1154 m to 0.0790 m, representing a reduction of 31.5%; the northward standard deviation decreases from 0.1309 m to 0.1251 m, a reduction of approximately 4.4%; and the up channel standard deviation decreases from 0.1775 m to 0.1452 m, a reduction of approximately 18.2%.

These results indicate that, under the combined influence of severe GNSS interference and high-dynamic carrier motions, the window-based adaptive noise estimation method incorporating a forgetting factor effectively mitigates the limitations of traditional algorithms, which overly rely on historical information and struggle to track noise variations. Consequently, the filtering error distribution becomes more concentrated with smaller fluctuations, thereby enhancing the robustness of the algorithm.

**Figure 20:**
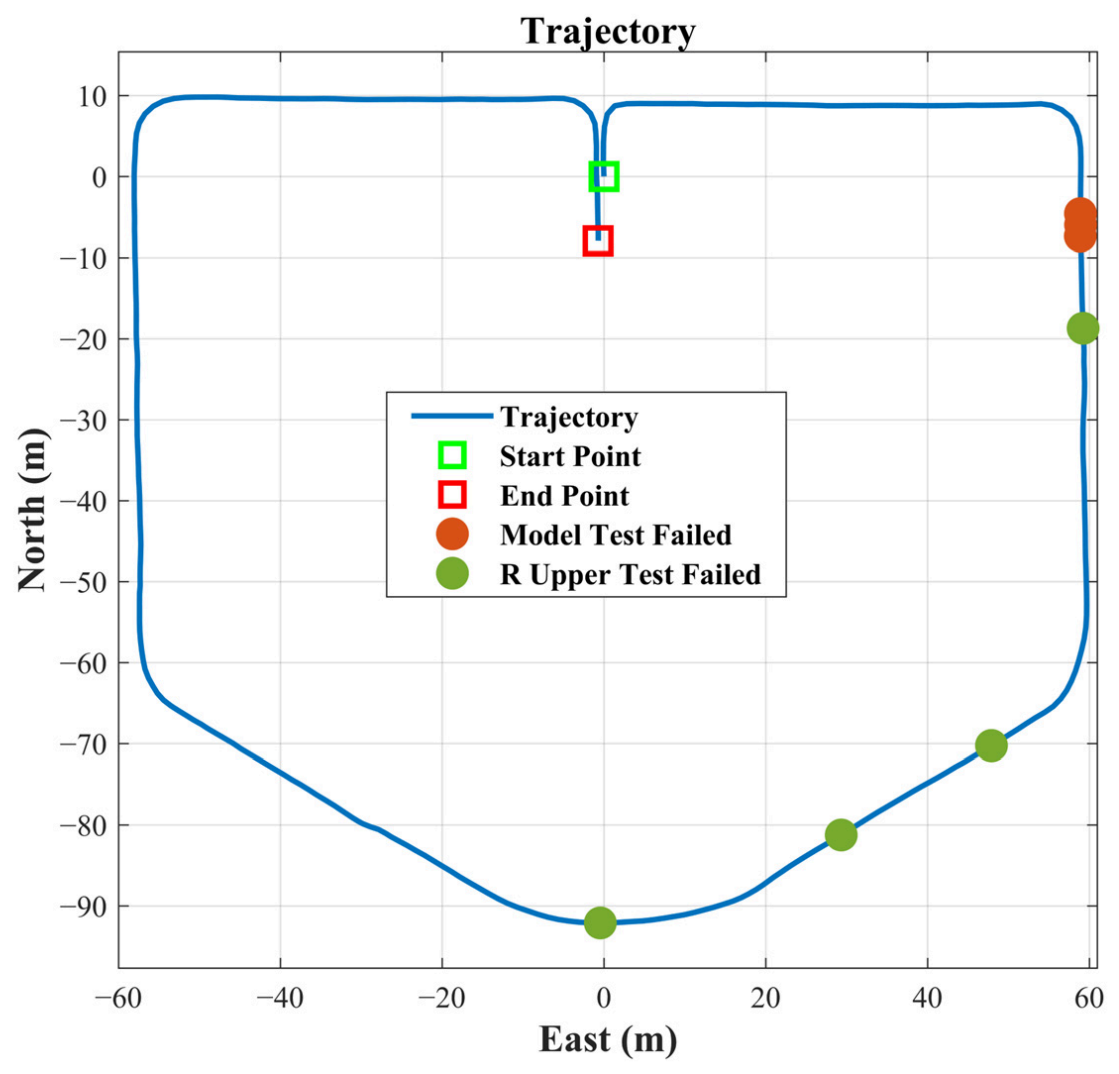
Out-of-Threshold Epochs Identified by the Model matching Test in Experiment 2.

As shown in Figure 18 and Figure 20, the carrier undergoes a 180° heading deflection after initialization. During this period, the east channel errors of both the cSage–Husa algorithm and the proposed iSage–Husa algorithm are slightly larger than that of the conventional EKF algorithm at the initial stage due to the influence of model mismatch. Subsequently, the model matching test module of the proposed algorithm identifies the abnormal amplification of the innovations induced by high-dynamic carrier motions. Under its coupled effect with the upper-bound test module, the east channel error is gradually suppressed. Approximately 130 s after initialization, the measurement noise estimation tends to stabilize, and the positioning accuracy begins to outperform that of the EKF algorithm. In contrast, the cSage–Husa algorithm is affected by the coupling of model mismatch and GNSS interference, and its measurement noise tracking estimation gradually becomes ineffective approximately 160 s after initialization, ultimately resulting in comprehensive error accumulation in the east, north, and up channels.

In summary, it can be observed that, under complex conditions where model mismatch and observation interference coexist, the proposed algorithm can effectively distinguish the impacts of these two error sources, and its observation covariance estimation achieves stable convergence.

**Figure 21:**
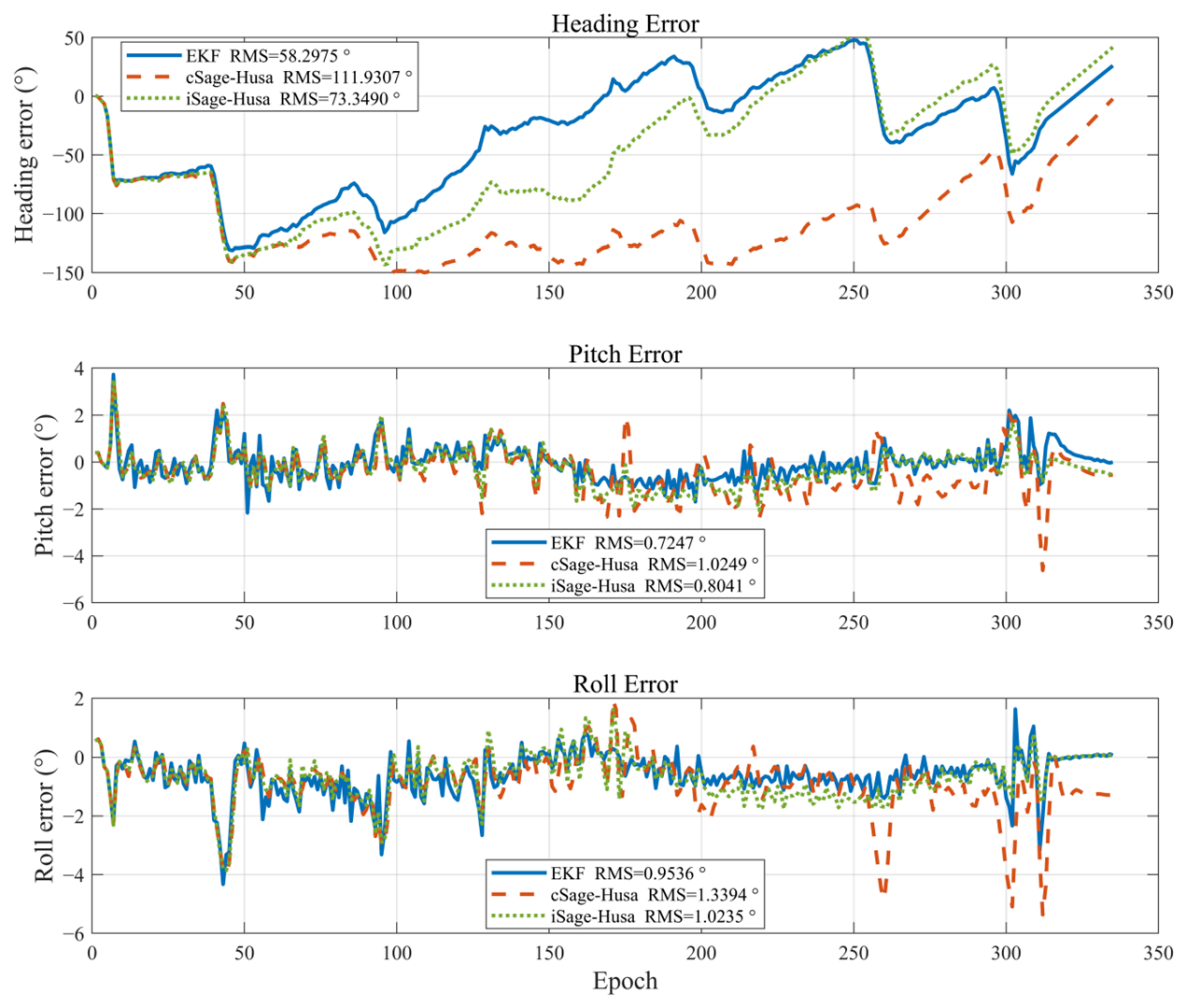
Attitude Error Analysis in Experiment 2.

(2) For attitude estimation (Figure 21), modeling errors in the INS kinematic model cause both Sage–Husa-based filters to overestimate the measurement noise covariance matrix during the initial stage, thereby resulting in slower convergence compared with the EKF. The iSage–Husa algorithm achieves faster convergence than the EKF after approximately 200 s, while the proposed method gradually exhibits superior estimation accuracy compared with the other two filters after approximately 250 s. Overall, the three filters demonstrate different attitude estimation performances under this test scenario.

Heading angle estimation is most significantly affected by the combined influence of high-dynamic carrier motions and GNSS signal interference. Owing to the lack of dynamic adaptability to severe motion states, the conventional cSage–Husa algorithm exhibits continuous error divergence under the coupled effects of model mismatch and measurement noise, indicating an evident deficiency in robustness. In contrast, by introducing a motion-state adaptation mechanism and a window adaptive scaling strategy, the proposed iSage–Husa algorithm effectively improves robustness against high-dynamic carrier motions, while maintaining adaptive estimation of measurement noise. The convergence speed of the error curves is significantly accelerated, and the errors can be rapidly suppressed after the recovery of observation conditions, demonstrating that the improved adaptive strategy substantially enhances the response capability to abrupt measurement noise variations and model mismatch. By comparison, the EKF algorithm exhibits better convergence in heading estimation; however, its performance in pitch and roll angle estimation is comparable to that of the adaptive filtering algorithms.

## 4. Discussion

Experimental results demonstrate that the proposed dual-adaptive Sage–Husa filtering algorithm incorporating a model matching mechanism effectively alleviates the over-adaptation and convergence degradation problems of conventional Sage–Husa algorithms under model mismatch conditions. It achieves a favorable balance between time-varying noise tracking capability and filtering stability, and exhibits superior overall performance across various complex navigation scenarios.

It is worth noting that, during the instantaneous GNSS outage and recovery process in Experiment 1, the up-channel RMS error of the iSage–Husa algorithm is reduced by 33.7% compared with the EKF, while that of the conventional Sage–Husa algorithm is reduced by approximately 36.7% relative to the EKF. This phenomenon can be attributed to the inherently lower positioning accuracy of GNSS in the up channel, which provides weaker constraints on the navigation solution. During the GNSS recovery phase, the iSage–Husa algorithm places greater emphasis on time-varying noise tracking and estimates the current measurement noise covariance adaptively, resulting in a slower convergence rate than approaches with excessive reliance on measurement information. This also explains why the proposed algorithm achieves better positioning performance in the east and north channels, where measurement accuracy is higher and system constraints are stronger.

In Experiment 2, under global GNSS interference conditions, all three algorithms are affected to different extents in heading angle estimation, with the EKF exhibiting a faster convergence rate. This phenomenon is mainly attributed to the fact that the vehicle undergoes two large-angle turns immediately after initialization, accompanied by multiple GNSS signal outages, which degrade the convergence performance of all filters. Moreover, insufficient heading angle estimation introduces model errors that contaminate the innovation sequence, thereby affecting the two Sage–Husa-based algorithms that estimate the measurement noise covariance from innovation statistics. In particular, such effects induce severe over-adaptation in the conventional Sage–Husa algorithm, resulting in accumulated model errors and further enlargement of the estimated measurement noise covariance, which significantly slows filter convergence and even causes a tendency toward divergence. In contrast, the proposed algorithm effectively suppresses this process through its adaptive mechanisms and achieves higher positioning accuracy after approximately 250 epochs, demonstrating improved capability in gross error identification and time-varying measurement noise tracking. It should be noted that once the filters enter the fully converged stage, the performance differences among different algorithms gradually diminish. Therefore, evaluating algorithm performance during the pre-convergence stage is of greater practical engineering significance, and the two experimental datasets in this study are processed independently. Although the EKF maintains an overall advantage in heading angle convergence speed, the proposed algorithm reduces the north-channel RMS error by 8.7% and 2.5% compared with the EKF and conventional Sage–Husa algorithm, respectively. Meanwhile, the up-channel RMS error is reduced by 15% compared with the conventional Sage–Husa algorithm while maintaining comparable accuracy to the EKF. The attitude estimation results demonstrate that the proposed algorithm effectively alleviates the over-adaptation and convergence degradation problems of conventional Sage–Husa algorithms under model mismatch conditions. Overall, the proposed algorithm enhances filtering robustness under complex dynamic motions and weak observation conditions, achieving a favorable balance between time-varying noise tracking capability and global filtering stability. Since the calculation interval mainly covers the period before complete filter convergence, the heading angle RMS error statistics remain relatively large; nevertheless, all algorithms exhibit an overall convergence trend.

Additionally, due to the limitations of the experimental conditions, all filtering algorithms were implemented using MATLAB R2021a offline scripts for post-processing, and no embedded real-time hardware platform was established. In an offline simulation environment, the execution time of MATLAB codes is affected by various factors unrelated to the intrinsic computational complexity of the algorithms, including interpreter efficiency, matrix operation optimization, and background process interference. Moreover, the underlying implementations of different algorithms were not optimized under a unified standard. Therefore, a direct comparison of single-epoch computational latency under offline conditions may not provide a fair evaluation and may not accurately reflect the real-time computational burden on onboard navigation processors. Accordingly, the computational time comparison among the three algorithms is not included in this study. In future work, real-time execution tests will be conducted to quantitatively evaluate the computational cost of each algorithm and further verify its engineering applicability for real-time vehicle navigation systems.

## 5. Conclusions

This paper proposes a model-matching-aware dual-adaptive robust filtering algorithm for GNSS/INS integrated navigation systems. The main conclusions are summarized as follows:

(1) Within the conventional Sage–Husa adaptive filtering framework, the proposed algorithm introduces an adaptive sliding window and a model matching test mechanism, establishing a dual-adaptive architecture that combines forgetting-factor-based sequential recursive updating with dynamic window adaptation. The proposed method effectively addresses the limitations of conventional algorithms, including the mismatch between the filtering model and carrier dynamic characteristics during the initial stage, insufficient responsiveness to rapidly time-varying noise, and vulnerability to innovation disturbances. Consequently, the rationality of noise estimation and the numerical stability of the filtering process are significantly improved.

(2) Experimental results demonstrate that, under initial high-dynamic motion and instantaneous GNSS interference conditions, the proposed algorithm reduces the overall up-channel RMS error by 8.5% compared with the conventional Sage–Husa algorithm. In locally GNSS-degraded regions, the up-channel RMS error is reduced by 33.7% compared with the EKF, while maintaining comparable accuracy to the conventional Sage–Husa algorithm. Under sustained GNSS interference conditions, the proposed algorithm reduces the north-channel RMS error by 8.7% and 2.5% compared with the EKF and conventional Sage–Husa algorithm, respectively. Meanwhile, the up-channel RMS error is reduced by 15% compared with the conventional Sage–Husa algorithm while achieving comparable performance to the EKF. These results indicate that the proposed method effectively alleviates the over-adaptation and convergence degradation problems of conventional Sage–Husa algorithms under model mismatch conditions.

(3) The proposed measurement noise covariance upper-bound constraint strategy and model matching test strategy can effectively identify and correct abnormal enlargement and excessive shrinkage of noise covariance estimates, thereby ensuring reliable and continuous operation of GNSS/INS integrated navigation systems under complex outdoor environments, high-dynamic carrier motions, and degraded observation conditions.

## Figures and Tables

**Table 1:** IMU Parameters.

	Specifications	ADIS16470	StarNeto XW-GI7660
Gyro	bias stability	8°/h	0.3°/h
	random walk	0.34°/$\sqrt{h}$	0.01°/$\sqrt{h}$
Acc	bias stability	1300 mGal	100 mGal
	random walk	0.037 m/s/$\sqrt{h}$	--

**Table 2:** Accuracy Metrics in Experiment 1.

Method	Position RMS (m)			Position MAX (m)			Position STD (m)		
	East	North	Up	East	North	Up	East	North	Up
EKF	0.2020	0.3176	0.6161	1.1038	0.4756	1.9975	0.1894	0.0737	0.5700
cSage–Husa	0.1985	0.3040	0.6299	1.0435	0.4894	1.5959	0.1906	0.1236	0.5750
iSage–Husa	0.1976	0.3160	0.5766	1.0365	0.5033	1.4947	0.1862	0.0792	0.5344

**Table 3:** Accuracy Metrics in Experiment 2.

Method	Position RMS (m)			Position MAX (m)			Position STD (m)		
	East	North	Up	East	North	Up	East	North	Up
EKF	0.2490	0.1863	0.3893	0.3823	0.3054	0.9134	0.0639	0.1328	0.1470
cSage–Husa	0.2296	0.1743	0.4564	0.4243	0.4132	0.8949	0.1154	0.1309	0.1775
iSage–Husa	0.2461	0.1700	0.3881	0.4208	0.2998	0.8931	0.0790	0.1251	0.1452

## Data Availability

The data used in this study are publicly available sample data and can be accessed at: https://github.com/GREAT-WHU/GREAT-FGO/tree/main/sample_data (accessed on 5 February 2026). No new independent dataset was created, and the raw GNSS observations, INS measurements, and related sample files used for algorithm validation are available through this public link.
